# Multivariate analyses to evaluate the contamination, ecological risk, and source apportionment of heavy metals in the surface sediments of Xiang-Shan wetland, Taiwan

**DOI:** 10.3389/fpubh.2025.1459060

**Published:** 2025-04-09

**Authors:** Ahmed Salah-Tantawy, Ching-Sung Gavin Chang, Shuh-Sen Young, Ching-Fu Lee

**Affiliations:** ^1^International Ph.D. Program in Environmental Science and Technology, University System of Taiwan (UST), Hsinchu, Taiwan; ^2^Institute of Analytical and Environmental Sciences, College of Nuclear Science, National Tsing Hua University, Hsinchu, Taiwan; ^3^Marine Science Division, Department of Zoology, College of Science, Al-Azhar University, Assiut, Egypt; ^4^Institute of Bioinformatics and Systems Biology, National Yang-Ming Chiao Tung University, Hsinchu, Taiwan

**Keywords:** sediments, ecological risk, Xiang-Shan, heavy metal, pollution index, sediment quality guidelines, wetlands, Taiwan

## Abstract

Nowadays, heavy metal (HM) contamination and their ecological risk in coastal sediments are global issues. This research provides insight into the heavy metals’ contamination, source apportionment, and potential ecological risks in the surface sediments of the Xiang-Shan wetland in Taiwan, which is undergoing rapid economic development, mainly by the semiconductor industries. The levels of twelve metals and total organic matter (TOM) were measured in 44 samples of surface sediment during the spring and winter seasons of 2022. Subsequently, the single and comprehensive pollution indices were assessed. The findings showed that the average of HM contents exhibited a descending sequence of Al > Fe > Mn > Zn > Co > Ga > Cr > Cu > In > Ni > Pb = Cd during both seasons. The E*_f_*, I*_geo_*, and PI showed that the majority of sediment samples were uncontaminated to heavily contaminated by Fe, Al, Zn, Cu, Mn, Cr, Ni, Co and Ga, and extremely contaminated by In. Moreover, PLI and mC*_deg_* unveiled that the surface sediments of DJ, OB, and KY stations were strongly or extremely polluted. PERI revealed that the sediment shows minimal to moderate ecological risk. The findings of multivariate analyses suggested that Fe, Al, Cu, Zn, and Ni derived from natural sources, while Ga, In, Co, Cr, and Mn originated from both anthropogenic and natural origins. Hence, it is critical that HM contamination, particularly Co, In, and Ga, be continuously monitored in the study area. Our data provide significant insights for more effective prevention and evaluation of HM contamination in the aquatic-sedimentary ecosystems of Taiwan.

## Introduction

Due to fast industrialization and urbanization, heavy metals (HMs) in marine ecosystems have been recognized as significant intermediary sources for the presence of contamination in marine environments and even population health (1). They are a grave hazard to people, living creatures, and natural settings owing to their unique physicochemical properties, such as high density, toxicity, persistence, bioaccumulation traits, and difficulty in removing them by self-purification (2–6). The accumulation of HM in living organisms and food webs is another manner in which HMs contribute to the deterioration of marine environments by diminishing species variety and richness (7, 8). Anthropogenically, HMs can enter marine and coastal ecosystems via multiple sources, e.g., agriculture, sewage, industries and household discharges (9). Additionally, they are triggered naturally by lithogenic events, including air deposition (10, 11).

Once heavy metals enter the aquatic system from different origins, some of them may dissolve, while others may bind to the suspended particles and eventually sink in the sedimentary substrate over time (12, 13). Due to fluctuations and discontinuities in water movement, sediment is an indispensable and dynamic factor in aquatic environments. It has biogeochemical and physical properties that assess the potential risks to the environment, and it has given us better tools for figuring out where heavy metals come from and how they are distributed than the water inspection over it (1). As a natural reservoir for the preponderance of metal contaminants dispersed into seawater, marine sediments can be utilized to evaluate the contamination level and environmental threat posed by HMs in various marine habitats (14–18). The evaluation of these characteristics offers crucial data about the effects of HM contamination and encourages environmentalists toward appropriate remediation solutions (19, 20). Likewise, such data will help authorities, legislators, and environmental activists understand the associations among coastal improvement and its efficient management to safeguard coastlines from global HM contamination (21).

Considering the vitality of the coastal ecosystem, several investigations on the contamination of sediments by HMs have been accomplished (22–26) and a number of geochemical and pollution indices, including the geoaccumulation index (I*_geo_*), contamination factor (*C_f_*), enrichment factor (E*_f_*), pollution load index (PLI), modified contamination degree (mC*_deg_*), potential ecological risk index (PERI), and sediment quality guidelines (SQGs), have been established in order to calculate the contamination level and environmental risk of HMs in marine sediments (25, 27–35). Furthermore, bivariate and multivariate statistical approaches, such as the Pearson’s correlation coefficient (PCC), Principle component analysis (PCA), and Hierarchical cluster analysis (HCA), are being implemented progressively to discover the potential origins of HMs and measure their pollution degree in sediments (26, 36–39).

In Taiwan, the government and researchers devoted scant attention to environmental issues spurred by sediment pollution with heavy metals. Recently, human operations for economic growth, mainly by industry, have been consistently and swiftly intensified, especially in Hsinchu city. After the 1980s, Hsinchu City had a new era of industrial expansion, and Hsinchu Science Industrial Park (HSIP) rose to the top position of semiconductor production around the globe. Besides, this park contained numerous innovative manufacturers of light-emitting diodes, liquid crystal displays, and optoelectronic plates, etc. According to the fabrication procedures of high-tech devices, a wide variety of substances are utilized in huge quantities. Despite stringent surveillance, the ultimate effluent water from the treatment plant still contains a significant proportion of contaminants (25). In HSIP, the daily water intake exceeds 200 thousand CMD, and the final wastewater from the wastewater treatment plant of the HSIP (over 100 thousand CMD) is released into the KeYa river. The Xiang-Shan wetland receives a large amount of freshwater from the KeYa stream since the KeYa river is the primary terrestrial source of freshwater. Over 40 % of freshwater production is wastewater from the treatment plant; approximately 40 % is untreated household waste; and less than 20 % is natural water gathered in the catchment region of the river. The new era of technological advancement introduced different forms of contaminants that settled on the surface of sediment and were immobilized by the adsorption process (40, 41). Therefore, it is critical to explore the ecological concerns and determine the existing level of HM pollution in marine sediments as well as the probable sources in Xiang-Shan wetland.

Yet, there is little accessible knowledge regarding the Xiang-Shan wetland’s heavy metal pollution and related health threats. Improving knowledge of sediment heavy metal pollution helps stakeholders, including the government and the public, safeguard the distinctive hydrological and biological ecosystem of the Xiang-Shan wetland. Therefore, 44 surface sediment samples were collected during two seasons to (1) investigate the sediment properties like, granulometric analysis (GSA) and total organic matter (TOM), (2) determine the total contents of twelve metals (e.g., Zn, Al, Ni, Fe, Cu, Mn, Co, Cr, In, Cd, Ga, and Pb), (3) assess the contamination level and possible risks associated with the studied elements, and (4) explore the potential origins of HMs by utilizing bivariate and multivariate statistical analysis. The findings on HM pollution and risks in the Xiang-Shan wetland’s surface sediments described herein are likely to be useful to environmental researchers and lawmakers.

## Materials and methods

### Study area

**Figure 1:**
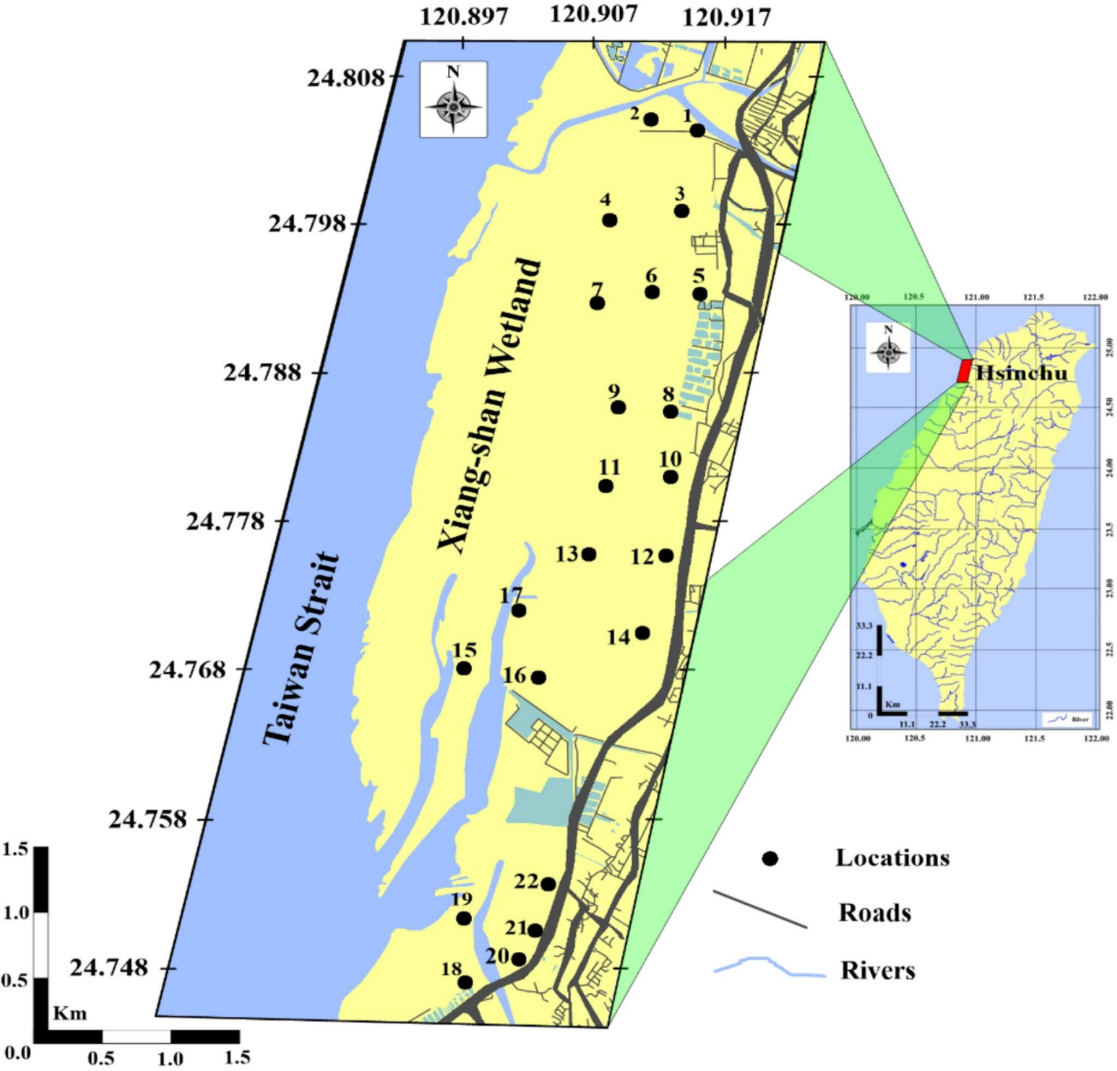
Map of the study stations illustrates the sampling locations (Surfer v. 10.7.972).

The Xiang-Shan wetland is situated west of Hsinchu city in Taiwan, between the KeYa river and HaiShan Fishing Harbor (Figure 1). The study area is 17 km^2^, with an 8-kilometer shoreline. It is characterized by fine sediments and a variety of species like crustaceans, prawns, benthic invertebrates, shellfish, and endangered avian species (42, 43). Since 1980, Hsinchu has been transformed into a significant center of high-tech industry, where the Hsinchu Science Industrial Park (HSIP) and its environs are home to the information technology (IT) industrial complex, commonly recognized as “Eastern Silicon Valley.” The HSIP is one of the largest emitters of treated water discharges (104,842 m^3^/d), according to Taiwan’s EPA permit registration. In the late 1990s, there were a number of noteworthy ecological incidents, such as the foul river water odor, aberrant blood test results of local residents, and frequent dead fish episodes in the KeYa stream (44, 45). The KeYa River is the main river that runs across the industrialized urban area. In fact, the watershed contains over 500 manufacturing facilities, including factories for electroplating, glass, cement, paper, pulp mills, computer chip manufacturing, container assembly, dyeing, rubber production, chemical plants, fertilizer manufacturing, printing, and metallic analyzing. Nowadays, the KeYa River continues to be the primary water body in Hsinchu City for collecting various pollutants dumped from domestic drainage from the surrounding population, agricultural and industrial effluent, and possibly occasionally illicit disposal of unprocessed wastewater from propagated industries (46). As a result, the Xiang-Shan wetland receives all of the freshwater from the KeYa river, and all contaminants from urban, agricultural, and industrial uses either sink in the sediments or are swept away by the shifting tides to the Taiwan Strait. Among the many contaminants, anthropogenic metals are extremely mobile and bioavailable and can harm aquatic creatures and human populations (47, 48).

### Sediment sampling and preparation

The study area covers an area of about 1,600 hectares overall, with a shoreline of about 8 km and it was split up into nine primary stations (KeYa (KY), KeYa Water Supply Center (KW), DaJuang (DJ), HuiMin (HM), FongCin (FC), HaiShan (HS), Oyster Bed (OB), YenKan (YK), and Mangrove Area (MA)), each of which had a number of locations spaced approximately 400 meters apart that extended from the shore to the interior (perpendicular to the coast). The main sampling stations were divided into 2, 2, 3, 2, 2, 3, 3, 2, and 3 locations for KY, KW, DJ, HM, FC, HS, OB, YK, and MA, respectively (Figure 1). In this research, 44 samples of surface sediment (0–5 cm deep) were gathered from 22 locations in the spring and winter of 2022. The same approach was employed to gather sediment samples in the winter as in the spring (*n* = 22).

At each sampling location, surface sediments were collected in labeled plastic bags using a sanitized glass scraper in order to prevent possible cross-contamination, and each sample was obtained by combining four subsamples. Then, about 500 g of combined sediment subsamples were put in a sealed plastic bag to keep the sample clean, clearly marked, and immediately transferred to the laboratory in a cool container. In our lab, sediment samples were dried in a dust-free area. The semi-dried state, it was smashed using an unpolluted glass vessel and dried in the oven at 50°C for two hours to eliminate the moisture content. Once dry, we removed non-sediment impurities such as roots, shells, gravel, and other debris. Following this, each sediment sample was split into three groups as follows: 100 g for GSA, 20 g for TOM, and 50 g for HMs analysis, and preserved at room temperature in plastic bags until examination. For heavy metal and total organic carbon analyses, about 10 g of each dried sediment sample were disaggregated with agate mortar into very fine grains (< 0.063 mm).

### Geochemical analyses

#### Grain size analysis

Mechanical sieve methods were used to perform grain size analysis (GSA) for Xiang-Shan sediments (49). The particle-size fractions were differentiated by passing 100 grams of dried sediment through a stainless-steel sieve. Particle sizes were expressed using the phi scale (*Φ*), since the logarithmic scale is more convenient than the equimultiple scale. Seven categories were acquired: gravel (*Φ*_−1_ > 2000 μm), very coarse sand (Φ_0_ > 1,000 μm), coarse sand (Φ_1_ > 500 μm), medium sand (Φ_2_ > 250 μm), fine sand (Φ_3_ > 125 μm), very fine sand (Φ_4_ > 63 μm) and silt or clay (Φ_5_ < 63 μm). The resultant sediment categories were re-classified into three distinct classes: gravel (Φ_−1_), sand (Φ_0_ + Φ_1_ + Φ_2_ + Φ_3_), and mud (Φ_4_ + Φ_5_) (25).

#### Total organic carbon

The Walkley-Black procedure was employed for quantifying total organic carbon (TOC) in surface sediments (50). 0.5 g of pulverized sediment was heated exothermically and oxidized with 1 N potassium dichromate (Cr_2_O_7_^−2^) and sulfuric acid (1:2). To eradicate excess dichromate, the solution was then adjusted with 0.5 N ferrous sulfate heptahydrate (FeSO_4_. 7H_2_O) solution after adding o-phenanthroline indicator (3 to 4 droplets). Accordingly, the results were multiplied by 1.8 to get the organic matter values. Likewise, the blank titration was carried out to standardize the Cr_2_O_7_^−2^.

#### Bioavailable of heavy metals concentrations (mg/kg)

Twelve metals were measured in surface sediment samples using the acid digestion method (51). To evaluate the heavy metal contents, 0.5 gram of each homogenized sample was digested by a 12 mL combination of hydrochloric and nitric acids (1:3) and then heated inside a microwave oven (MarsXpress) for 12 min at 175°C. After the digestion process, each extract was dissolved into fifty milliliters of high-purity water (Millipore Direct-Q System), filtrated by filter paper with a pore size of 40 mm (ADVANTEC, Japan), and Zn, Al, Ni, Fe, Cu, Mn, Co, Cr, In, Cd, Ga, and Pb concentrations were measured using an inductively coupled plasma (ICP-OES) at National Tsing Hua University in Taiwan. The ICP multi-element standard solution (1,000 ppm) was employed to generate the calibration curves, and the samples were only examined when the *r^2^* was higher than 0.995. The instrument was recalibrated if there was a deviation of over 10% after the initial calibration and after the analysis of ten samples. Also, the recovery rates for the examined heavy metals fluctuated between 96.3 and 103%. For quality control, all apparatus was cleaned and sterilized for 24 h in a nitric acid solution (10%) before being rinsed in double-distilled water. In our research, Merck PA reagents were employed throughout the experiments. The results were displayed as mg/kg and three digestions of each sample were achieved.

### Determination of pollution degree

#### Single pollution indices

##### Enrichment factor (E_f_)

To assess the level of HM enrichment in sediment, the E*_f_* was applied by comparing the measured element to a reference metal (52).

In our work, Iron (Fe) served as a conservative element to standardize the detected metal levels in sediment because it is the fourth most prevalent element in the shale, has a natural content that tends to be consistent, is a carrier of numerous metals, and has a fine uniform surface (53, 54). Ef values are given by the following formula (55):

$$Ef^{i}=\left(C_{m}^{i}÷{Fe}_{m}\right)\mathrm{Sample}/\left(C_{b}^{i}÷{Fe}_{b}\right)\mathrm{crust}$$

Where $C_{m}^{i}$ and $C_{b}^{i}$ are the ratios of the sample’s heavy metal *i* value to its earth’s crust value, respectively; whereas ${Fe}_{m}$ and ${Fe}_{b}$ are the detected iron level and its value in the crust, respectively. Here, we used the average shale values (ASVs) determined by Turwkian and Wedepohl as the reference, which are: Zn: 95, Al: 80,000, Ni: 68, Fe: 47,200, Cu: 45, Mn: 850, Pb: 20, Cr: 90, In: 0.1, Cd: 0.3, and Co = Ga: 19 mg.kg^−1^ (56). Since the enrichment factor technique does not have a recognized classification scheme for contamination levels, seven professional classes have been offered in Table 1 (57).

**Table 1:** Degrees of heavy metal contamination determined by single pollution indices.

Categories	E*_f_*	Contamination degree	I*_geo_*	Contamination degree	*C_f_* / PI	Contamination degree
0	< 1	No enrichment	< 1	Nil to minor pollution	*C_f_* < 1	Low pollution
1	1 ≤ E*_f_* < 3	Minor enrichment	1 ≤ I*_geo_* < 2	Moderate pollution	1 ≤ *C_f_* < 3	Moderate pollution
2	3 ≤ E*_f_* < 5	Moderate enrichment	2 ≤ I*_geo_* < 3	Severe pollution	3 ≤ *C_f_* < 6	Considerable pollution
3	5 ≤ E*_f_* < 10	Heavily enrichment	3 ≤ I*_geo_* < 4	Very severe pollution	*C_f_* > 6	High pollution
4	10 ≤ E*_f_* < 25	Severe enrichment	4 ≤ I*_geo_* < 5	Significant pollution		
5	25 ≤ E*_f_* < 50	Very severe enrichment	I*_geo_* > 5	Extreme pollution		
6	E*_f_* > 50	Extremely enrichment				
Acevedo-Figueroa et al. (57)			Förstner et al. (124)		Chakraborty et al. (62) and Tian et al. (109)	

##### Geo-accumulation index (I*_geo_*)

The geoaccumulation index is applied to calculate the HMs contamination without taking into consideration geogenic conditions (58). I*_geo_* can be calculated as follows:

$${\mathrm{Igeo}}^{i}=\log_{2}\left(C_{m}^{i}÷1.5\;C_{b}^{i}\right)$$

wherein 1.5 represents the baseline matrix adjustment factor that mitigates the influences of geological contributions (59, 60). Based on the I*_geo_*, sediment samples can be allocated into different distinct categories (Table 1).

#### Comprehensive pollution indices

##### Pollution load index (PLI)

PLI is calculated as the *n*th root of the outcome of *n C_f_* and can be used to ascertain the aggregate pollution at the studied stations. The subsequent equations were employed to compute $C_{f}^{i}$ and PLI (61, 62):

$$C_{f}^{i}=\left(C_{m}^{i}∕C_{b}^{i}\right)$$

$${PLI}^{i}=\sqrt[{n}]{\left(\begin{array}{l} Cf_{Fe}\times Cf_{Al}\times Cf_{Mn}\times Cf_{Zn}\times Cf_{Cu}\times \\ Cf_{Ni}\times Cf_{Co}\times Cf_{Cr}\times Cf_{\mathrm{Ga}}\times Cf_{\mathrm{In}} \end{array}\right)}$$

whereas $C_{f}^{i}$ refers to the single contamination factor for the metal *i*. As shown in Tables 1, 2, the $C_{f}^{i}$ and PLI have been classified into several pollution levels.

**Table 2:** Categories of heavy metal pollution by comprehensive pollution indices.

Classes	PLI	Pollution level	mC*_deg_*	Pollution level	P*_N_*	Pollution level
1	< 1	Unpolluted	< 2	Nil to very low contamination	< 0.7	Non-polluted
2	1 ≤ PLI < 2	Slightly polluted	2 ≤ mC*_deg_* < 4	Slight contamination	0.7 ≤ P*_N_* < 1	Minor pollution
3	2 ≤ PLI < 3	Strongly polluted	4 ≤ mC*_deg_* < 8	Strong contamination	1 ≤ P*_N_* < 2	Moderate pollution
4	PLI ≥ 3	Heavily polluted	8 ≤ mC*_deg_* < 16	Heavy contamination	2 ≤ P*_N_* < 3	Significant pollution
5			16 ≤ mC*_deg_* < 32	Severe contamination	P*_N_* > 3	Extremely pollution
6			mC*_deg_* > 32	Extremely contamination		
Tian et al. (62)			Abrahim and Parker (63)		Yang et al. (65)	

##### Modified contamination factor (mC*_deg_*)

Likewise, the comprehensive pollution of multiple elements per sampling station was evaluated utilizing the modified degree of contamination (mC*_deg_*) approach (30). mC_deg_ developed by Abrahim and Parker (63) and it calculated as follows:

$$C_{f}^{i}=\left(C_{m}^{i}∕C_{b}^{i}\right)$$

mCdeg=∑Cfin

Since *n* indicates the number of measured elements. The mC*_deg_* is classified into various classes; see Table 2.

###### Nemerow comprehensive pollution index (P*_N_*)

The Nemerow comprehensive pollution index (P*_N_*) is another method for determining the total pollution degree of heavy metals throughout all stations (64), and it was estimated using an individual pollution index (PI):

$$\mathrm{PI}=\left(C_{m}^{i}∕C_{b}^{i}\right)$$

$$P_{N}=\sqrt{\frac{{\left({\mathrm{PI}}_{ave}^{i}\right)}^{2}+{\left({\mathrm{PI}}_{\text{max}}^{i}\right)}^{2}}{2}}$$

Where ${\mathrm{PI}}_{ave}^{i}$ represents the average singular pollution index level of a metal, and ${\mathrm{PI}}_{\text{max}}^{i}$ signifies its maximum level. Based on Yang et al. (65), P*_N_* is categorized into five levels of pollution (Table 2).

#### Evaluate the potential ecological risk

##### Potential ecological risk index (PERI)

This study applied the PERI in order to evaluate the possible risks posed by heavy metals (66). This index extensively considers the synergy, hazardous threshold, heavy metal content, and environmental sensitivity of elements (67–69). The PERI is composed of three fundamental factors: potential ecological risk factor ( $E_{R}^{i}$ ), toxic-response factor ( $T_{r}^{i}$ ), and contamination level ( $C_{m}^{i}$ ). Accordingly, both individual ( $E_{R}^{i}$ ) and cumulative ( PERI ) ecological risks were computed via these equations:

$$C_{f}^{i}=\left(C_{m}^{i}∕C_{b}^{i}\right)$$

$$E_{R}^{i}=T_{r}^{i}\;x\;C_{f}^{i}$$

$$\mathrm{PERI}=\overset{m}{\underset{i=1}{\sum }}E_{R}^{i}$$

Where $C_{f}^{i}$ and $E_{R}^{i}$ reflect to the single contamination factor and potential ecological risk index for the element *i*, respectively, while $T_{r}^{i}$ is the biological toxic factor of a certain metal that is established for Mn = Zn = 1, Cd = 30, Cr = 2, and Cu = Ni = Co = Pb = 5 (66).

In this research, eight contaminants involving Zn, Ni, Cr, Pb, Mn, Co, Cu, and Cd are considered by the classical PERI method. The current work modified the classification guideline for the metal’s ecological risk indices as a result of the variation in contaminant forms and quantities (70). The greatest value of $T_{r}^{i}$ had been chosen to represent the minimal level limit of $E_{R}^{i}$ , and the subsequent level limits were then doubled. Similarly, by setting the rounding digit of $\sum T_{r}^{i}$ as the smallest level limit of PERI, the subsequent level limits were then doubled (70). The modified classification guidelines of PERI in sediment are illustrated in Table 3.

**Table 3:** Classification of ecological risks posed by heavy metal pollution.

Classes	E*_R_*	PERI	Single and comprehensive ecological risk level
1	< 30	< 40	Minimal risk
2	30 ≤ E*_R_* < 60	40 ≤ PERI <80	Moderate risk
3	60 ≤ E*_R_* < 120	80 ≤ PERI <160	Considerable risk
4	120 ≤ E*_R_* < 240	160 ≤ PERI <320	Strong risk
5	E*_R_* > 240	PERI >320	Extremely risk
Hakanson (66) and Li et al. (70)			

##### Sediment quality guidelines (SQGs)

Our findings were compared with different worldwide guidelines to better express the quality and adverse effects of HMs in sediment. This method includes four international guidelines: (1) the Australian and New Zealand Environment and Conservation Council and the Agriculture and Resource Management Council of Australia and New Zealand (71); (2) the National Oceanic and Atmospheric Administration of the USA (NOAA) (72); (3) the Canadian Council of Ministers of the Environment (73); and (4) Taiwan’s national standard guidelines (74). For different heavy metals, there are lower and upper limits for each of the four typical guidelines. Negative effects “infrequently or rarely emerge” if the metal level surpasses the lower limit, but they “frequently occur” if the level surpasses the upper limit (75).

### Statistical analyses

The data were pre-processed utilizing the Excel Pro +2019 software, and they are demonstrated as averages for the studied locations. All descriptive data (e.g., maximum, minimum, average, and standard deviation) and the ANOVA were executed by SPSS version 25 (*p* < 0.05) (76, 77). In order to compute the HMs pollution and their probably risks in the sediment of the Xiang-Shan wetland, several ecological pollution indicators were computed and visualized by Origin 2021 (v. 9.8). Simultaneously, multivariate statistical analyses such as principal component analysis (PCA) and hierarchical cluster analysis (HCA) were conducted to identify probable heavy metal sources (78). Furthermore, the relationship among HMs and sediment properties was examined via Pearson’s correlation coefficient (PCC) to validate the findings of multivariate analyses (26). PCC and PCA were displayed using the “*corrplot*” package in R programming v. 4.2.2 (*p* < 0.001, 0.01, and 0.05) (79, 80) and Origin 2021 (v. 9.8), respectively, while the HCA dendrogram was plotted by the PC-ORD 5 program (81) according to the Euclidean distance and the Ward methods.

## Results

### Sediment properties

#### Grain size analysis

**Figure 2:**
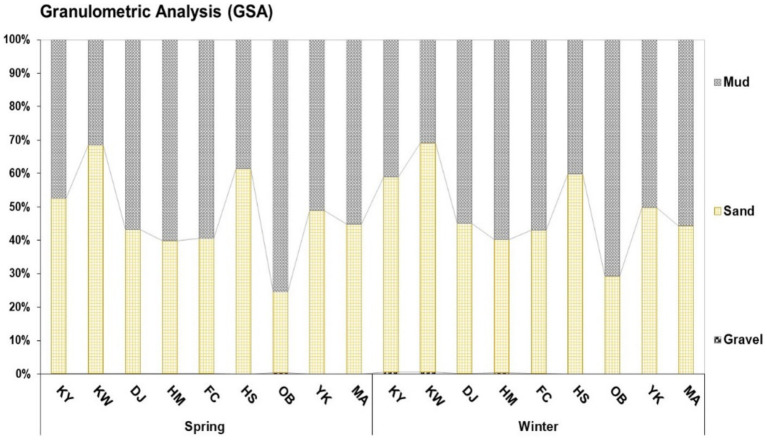
Grain size analysis of surface sediments at Xiang-Shan wetland during the spring and winter seasons (KY: KeYa, KW: KeYa Water Supply Center, DJ: DaJuang, HM: HuiMin, FC: FongCin, HS: HaiShan, OB: Oyster Bed, YK: YenKan, MA: Mangrove Area).

The granulometry of surface sediment at Xiang-Shan wetland is depicted in Figure 2. Based on the GSA findings, sediment grains were divided into seven fractions with various sizes and further grouped into three major classes (gravel, sand, and mud). Seasonally, the mean particle size of surface sediment fluctuated between (0.00–0.38%) for gravel, (24.18–68.44%) for sand, and (31.35–75.44%) for mud in spring, while in winter it ranged from 0.03 to 0.51%, 29.10 to 68.64%, and 30.86 to 70.84% for gravel, sand, and mud, respectively. Geographically, the surface sediments of KY, KW, and HS stations were predominated by sand, while DJ, HM, FC, OB, and MA were characterized by mud sediments in both seasons. Overall, all studied stations were dominated by mud and sand sediments. In contrast, the gravel particles demonstrated minimal proportions at all stations.

#### Total organic matter (TOM)

**Figure 3:**
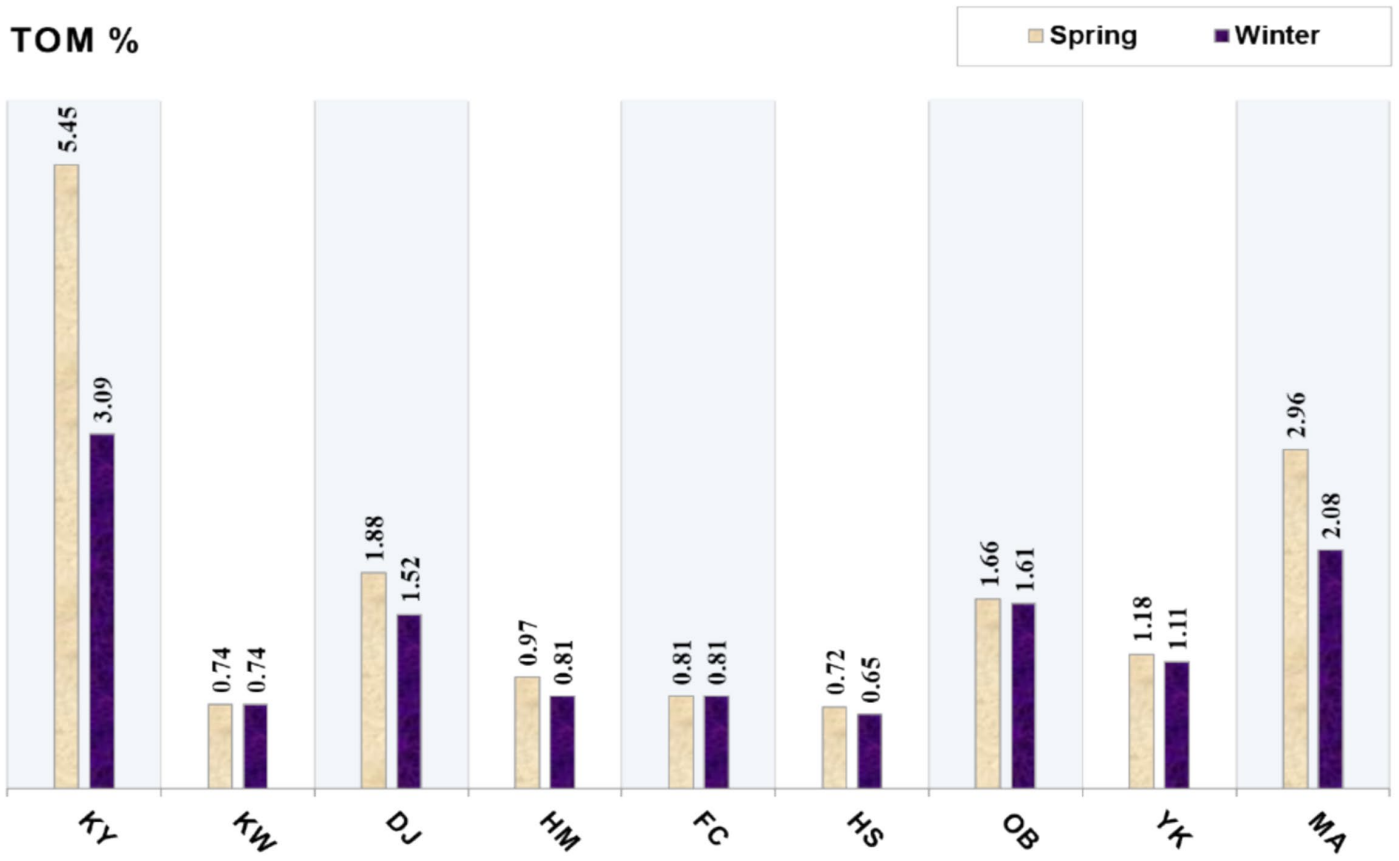
The mean contents of total organic matter in the Xiang-Shan wetlands’ sediments during both seasons (KY: KeYa, KW: KeYa Water Supply Center, DJ: DaJuang, HM: HuiMin, FC: FongCin, HS: HaiShan, OB: Oyster Bed, YK: YenKan, MA: Mangrove Area).

The mean TOM contents in the surface sediment of the Xiang-Shan wetland are illustrated in Figure 3. The TOM levels at the surface sediments fluctuated between 0.72–5.45% and 0.65–3.09% in spring and winter, respectively. Moreover, the greatest content of TOM was recorded at KY station, followed by MA, OB, and DJ, while the lowest contents were recorded at HS station during different seasons. Specifically, the surface sediments of the KY and MA stations were highly enriched with TOM in both seasons.

### Total concentrations of HM in surface sediments

Supplementary Table 1 illustrates the average levels of HMs in the surface sediment of the examined stations during the two seasons. The levels of Iron (Fe), Aluminum (Al), Magnesium (Mn), Copper (Cu), Zinc (Zn), Gallium (Ga), Indium (In), Nickel (Ni), Chromium (Cr), Cobalt (Co) varied in the ranges of 24115.00–42123.33, 19234.50–51850.00, 319.15–764.73, 12.17–117.80, 65.90–252.05, 64.05–121.63, 27.40–56.23, 15.55–45.25, 55.35–112.87, 69.25–134.60 mg/kg, respectively, for spring, and 23445.00–38624.67, 20785.00–48285.00, 266.05–667.37, 11.05–77.15, 60.20–233.80, 57.35–106.40, 17.90–48.57, 16.90–37.05, 46.90–109.23, 58.35–121.97 mg/kg, respectively, for winter. All stations had Pb and Cd concentrations below the detection limit for both seasons. Spatially, the maximum levels of HMs such as Al, Co, Cr, Ga, and In at DJ station (51850.00, 134.60, 112.87, 121.63, and 56.23 mg/kg, respectively) were observed in the spring season, while Zn, Cu, and Ni were detected at KY station (252.05, 117.80, and 45.25 mg/kg, respectively). OB and MA stations recorded high concentrations of Mn and Fe (764.73 and 42123.33, respectively). Inversely, surface sediments at KW station exhibited the minimum levels of Fe, Al, Co, Cr, and Ga (23445.00, 19234.50, 58.35, 46.90, and 57.35 mg/kg, respectively), and at YK station for Zn, Ni, and In (60.20, 15.55, and 17.90 mg/kg, respectively). Also, Mn and Cu concentrations (266.05 and 11.05 mg/kg) were low in KY and FC stations respectively, in the winter.

### Assessment of heavy metal- polluted sediments

In our research, five reliable indicators were employed to estimate the extent of contamination by HMs in surface sediments, of which two indicators (E*_f_* and I*_geo_*) were employed to gauge the pollution by certain metals, and the other three (PLI, mC*_deg_*, and P*_N_*) were used for comprehensive pollution assessment.

#### Enrichment factor (E*_f_*)

**Figure 4:**
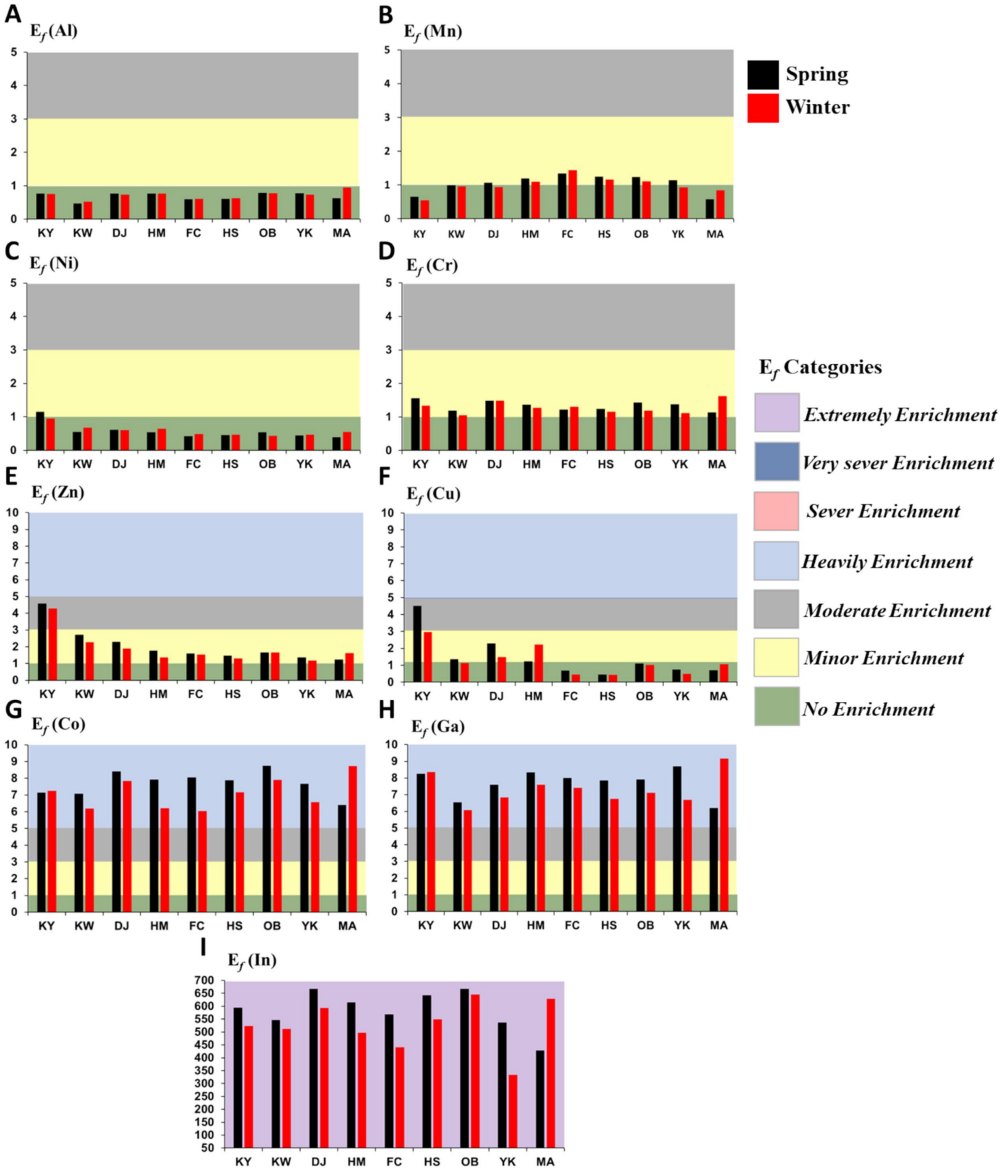
Enrichment factor (Ef) and contamination levels for studied HMs in the XiangShan wetland’s sediments. (KY: KeYa, KW: KeYa Water Supply Center, DJ: DaJuang, HM: HuiMin, FC: FongCin, HS: HaiShan, OB: Oyster Bed, YK: YenKan, MA: Mangrove Area). **(A)** Al, **(B)** Mn, **(C)** Ni, **(D)** Cr, **(E)** Zn, **(F)** Cu, **(G)** Co, **(H)** Ga, **(I)** In.

Figure 4 depicts the calculated E*_f_* and contamination degree for each metal in Xiang-Shan wetland based on Iron (Fe) as the reference metal. The ranges (mean) of the E*_f_* of Al, Mn, Cu, Zn, Co, Cr, Ni, Ga, and In at the study area during different seasons were 0.47–0.94 (0.70), 0.54–1.43 (1.02), 0.43–4.51 (1.35), 1.18–4.57 (1.99), 6.05–8.75 (7.40), 1.05–1.62 (1.31), 0.39–1.15 (0.58), 6.08–9.17 (7.52), and 334.00–666.93 (554.85), respectively. Based on the categories of E*_f_* (Table 1), Al, Cd, and Pb showed no enrichment (class 0) in all study stations, while Zn, Cu, Ni, and Cr fluctuated between class 0 (< 1) and class 2 (< 5). Moreover, E*_f_* for Co and Ga were subjected to class 3 (< 10), and class 6 for In (> 50).

#### Geoaccumulation index (I*_geo_*)

**Figure 5:**
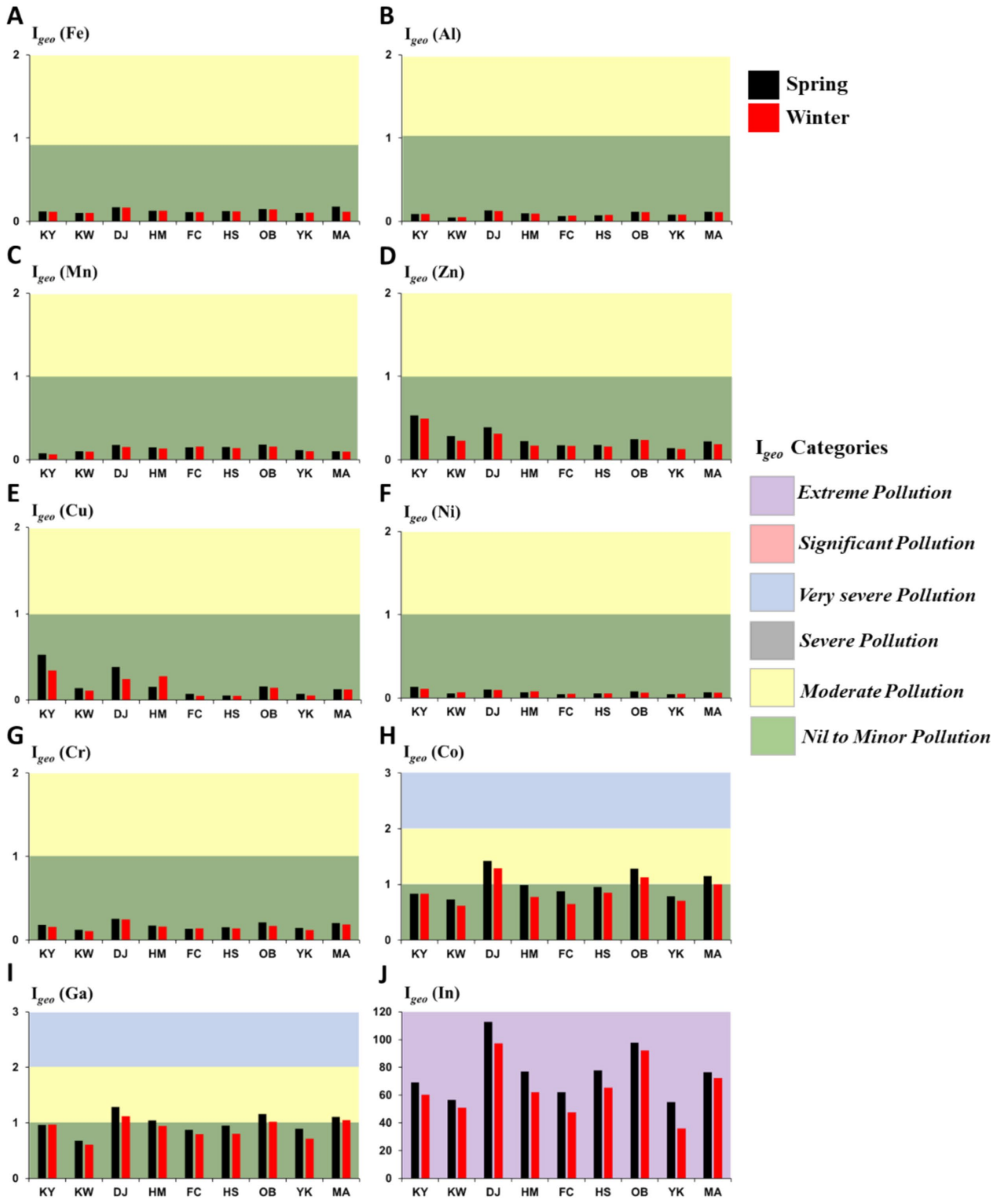
Geoaccumulation index (I_geo_) and contamination categories for analyzed HMs in the Xiang-Shan wetlands’ sediments (KY: KeYa, KW: KeYa Water Supply Center, DJ: DaJuang, HM: HuiMin, FC: FongCin, HS: HaiShan, OB: Oyster Bed, YK: YenKan, MA: Mangrove Area). **(A)** Fe, **(B)** Al, **(C)** Mn, **(D)** Zn, **(E)** Cu, **(F)** Ni, **(G)** Cr, **(H)** Co, **(I)** Ga, **(J)** In.

The computed I*_geo_* and contamination degree at all sampling stations in two seasons are demonstrated in Figure 5. The ranges (mean) of the I*_geo_* of Fe, Al, Mn, Cu, Zn, Ni, Co, Cr, Ga, and In were 0.10–0.18 (0.13), 0.05–0.13 (0.09), 0.06–0.18 (0.13), 0.05–0.53 (0.17), 0.13–0.53 (0.25), 0.05–0.13 (0.07), 0.62–1.42 (0.94), 0.10–0.25 (0.17), 0.61–1.28 (0.94), and 35.92–112.84 (70.54), respectively. According to Table 1, the I*_geo_* levels for all investigated metals at all studied stations in the spring and winter were unpolluted (< 1), except Co and Ga at DJ, OB, and MA stations showed moderate pollution (class 1), and In values subjected to class 5 (> 5) at all study stations.

#### Pollution load index (PLI)

**Figure 6:**
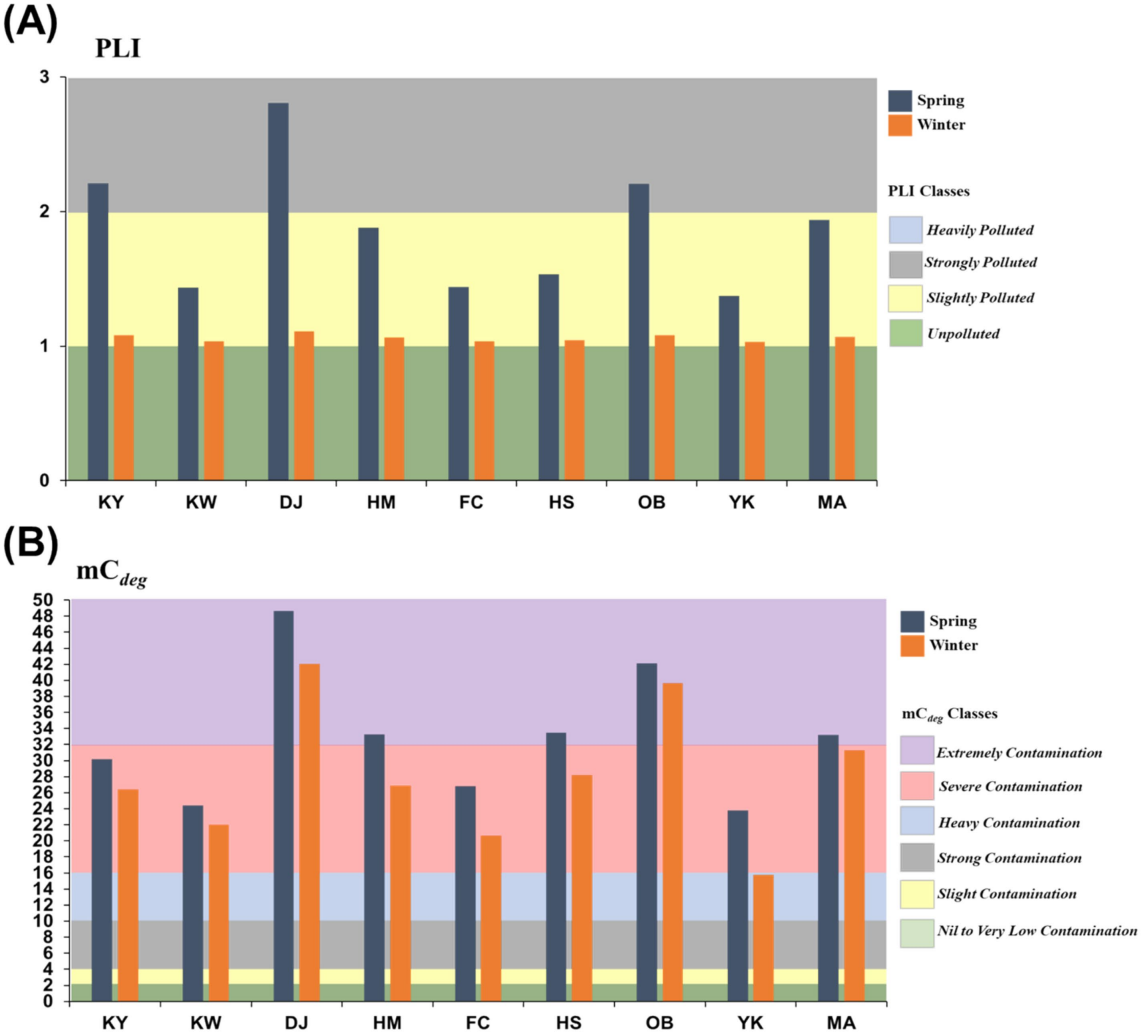
Comprehensive pollution indices with pollution classes at the studied stations. **(A)** Pollution load index and **(B)** Modified contamination degree (mC*_deg_*) (KY: KeYa, KW: KeYa Water Supply Center, DJ: DaJuang, HM: HuiMin, FC: FongCin, HS: HaiShan, OB: Oyster Bed, YK: YenKan, MA: Mangrove Area).

The PLI, as a comprehensive index, served to measure the deposition levels of HMs in the Xiang-Shan wetland’s surface sediments, as shown in Figure 6A. Seasonally, PLI varied from 1.37 to 2.80 and 1.03 to 1.11 with an average of 1.87 and 1.06 in the spring and winter samples, respectively, indicating the range of slightly polluted (< 1) to heavily polluted (> 2). The mean values of PLI at most studied stations fall under class 1 (1 ≤ PLI < 2) in both spring and winter sediment samples, except at 3 stations (KY, DJ, and OB) where they were greater than 2 (class 2) in the spring season (Table 2).

#### Modified contamination degree (mC*_deg_*)

Modified degree of contamination (mC*_deg_*) was implemented to compute the overall pollution level of all HMs in surface sediment samples (Figure 6B). mC*_deg_* varied from 23.82 to 48.65 with an average of 32.88 and from 15.77 to 42.03 with an average of 28.10 in the sediments during spring and winter, respectively. According to the mC*_deg_* classification (Table 2), the sampling stations fluctuated between severe and extremely polluted (classes 5 and 6, respectively) in the spring season, while, in the winter, the contamination degree ranged from heavy to extremely (classes 4 and 6, respectively) at the studied stations.

#### Nemerow integrated pollution index (P*_N_*)

The P*_N_* index was calculated to calculate the comprehensive contamination for each metal across all sediment samples; see Supplementary Table 2. In our study, the mean levels of P*_N_* for each metal ranged from 0.00 to 479.66 with average (38.72), reflecting the range of unpolluted (P*_N_* < 0.7) to extremely polluted (P*_N_* > 3). The mean P*_N_* values for In, Ga, and Co were subjected to class 4 (P*_N_* > 3), while the other metals ranged from unpolluted (P*_N_* < 0.7) to significant pollution (2 ≤ P*_N_* < 3).

### Determine the potential ecological risks of heavy metals

#### Potential ecological risk index (PERI)

**Figure 7:**
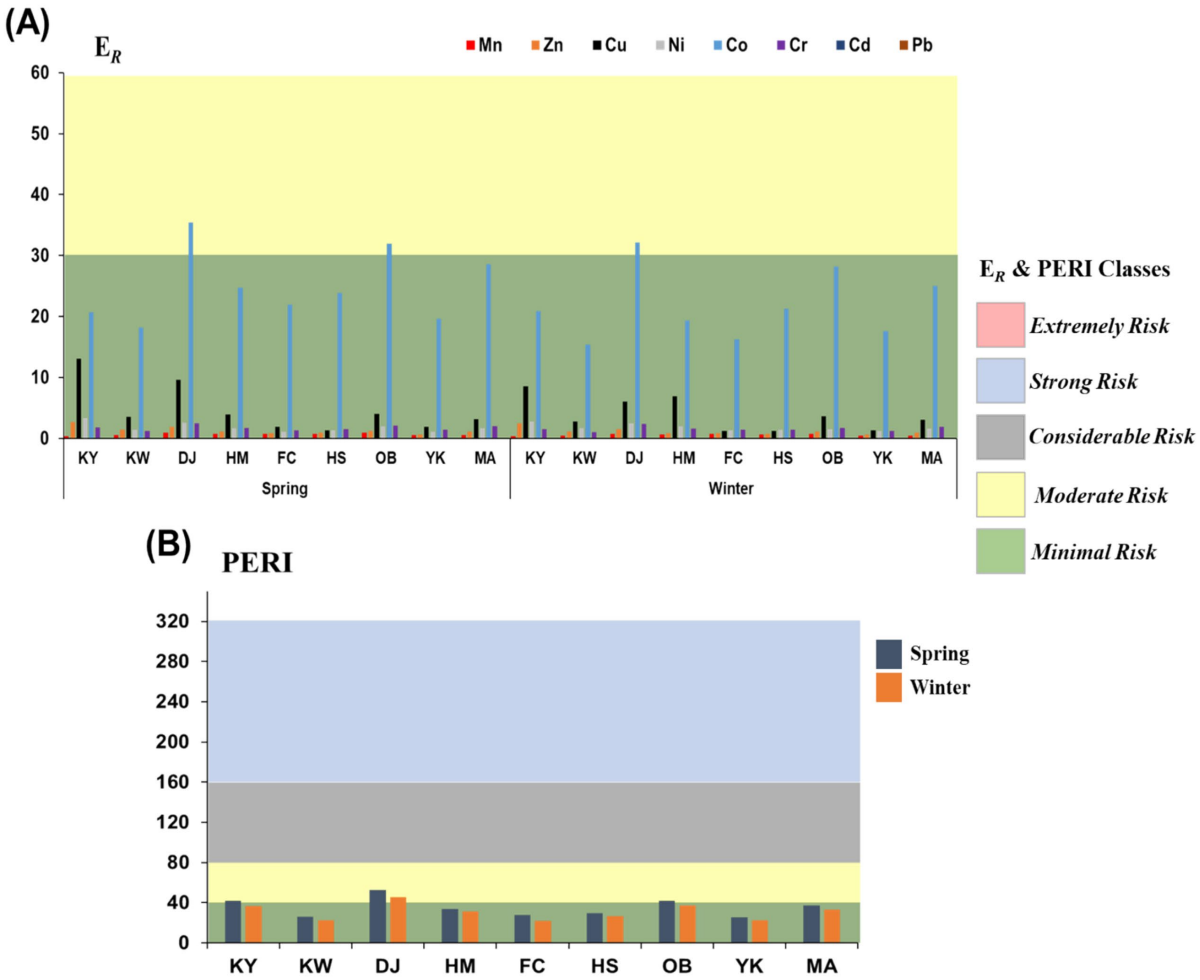
Potential ecological risk index with pollution classes for the studied HMs. **(A)** Single ecological risk (E*_R_*) for each metal and **(B)** Integrated potential ecological risk index (PERI) (KY: KeYa, KW: KeYa Water Supply Center, DJ: DaJuang, HM: HuiMin, FC: FongCin, HS: HaiShan, OB: Oyster Bed, YK: YenKan, MA: Mangrove Area).

PERI is an integrative indicator that is calculated from the individual ecological risk (E*_R_*) of each element. As shown in Figure 7A and Table 3, the mean E*_R_* values for Cr, Mn, Cu, Zn, Pb, Cd, and Ni fall under class 1 (E*_R_* < 30), while for Co ranged between minimal (E*_R_* < 30) and moderate risk (30 ≤ E*_R_* < 60) throughout the Xiang-Shan wetland in both spring and winter. Additionally, PERI ranged between 25.32–52.97 (mean = 35.25) in summer and 21.77–45.37 (mean = 30.74) in winter (Figure 7B). Based on the PERI classes (Table 3), all studied stations displayed a minimal ecological risk to the environment, with PERI levels below 40 (class 1), except KY and OB stations in the spring and DJ in both seasons (class 2, > 40).

## Discussion

### Sediment characteristics

Granulometric analysis was performed mechanically to differentiate the surface sediment particles of the Xiang-Shan wetland and consequently establish the accumulation trend of organic matter and HMs in relation to the sediment size. The results of GSA revealed that mud sediments are the dominant grain size overall, followed by sand sediments (*p* = 0.07), and gravel (*p* = 0.003) across all sampling stations with averages (51.98, 47.82, and 0.19%, respectively). Specifically, DJ, HM, FC, OB, and MA stations were dominated by mud, while sand grains were dominant in KY, KW, and HS stations. Overall, the surface sediments of the Xiang-Shan wetland were characterized by fine-grained sediments (mud and sand). This may be attributed to flow rate, flow velocity, and calm conditions. Rea and Hovan (82) reported that fine-grained sediments are conveyed by suspension in the marine setting; therefore, they can readily be distributed throughout the water mass and transported for long distances before being re-deposited in the calm zone. The percentages of GSA among the studied stations decreased in the order of mud > sand > gravel.

Sediment organic matter is composed of light-weight materials, typically structural materials from marine creatures (83). In our study, KY station had the maximum TOM content with an average of 4.27%, followed by MA (2.52%) in both seasons. This could be attributed to the KeYa River, which supplies the KY station with an extensive amount of freshwater loaded with a high proportion of OM (25). Furthermore, the mangrove environment is regarded as a highly productive ecosystem with substantial rates of organic matter storage (84, 85). Also, DJ and OB stations had relatively significant values, with averages of 1.70 and 1.64%, respectively; this may be associated with the deposition of fine particles with excessive organic matter levels. The magnitude of TOM values among the surface sediments of Xiang-Shan wetland were in the order of KY > MA > DJ > OB > YK > HM < FC > KW > HS for both spring and winter seasons. The ANOVA revealed insignificant spatio-temporal variances in TOM (*p* > 0.05).

### Heavy metal concentrations and comparison with worldwide studies

Heavy metals are pervasive and tenacious in marine settings, probably poisonous, and may be accumulated in food chains (86, 87). Multiple pathways, including air deposition, agriculture, and industrial activities, have been identified as the origins of HM contamination in sediments (88, 89). In our research, there was no discernible difference in the concentration of HMs between the spring and winter seasons (*p* > 0.05), despite an overall greater level observed during the spring season across all sampling stations. Higher heavy metal concentrations in the spring may be caused by seasonal changes in the wetland’s water flows; such as, water replenishment to the wetland is restricted in the spring, resulting in less mobility and greater deposition of HMs in surface sediments (90).

The average levels of Iron (Fe), Aluminum (Al), Manganese (Mn), Cobalt (Co), Zinc (Zn), copper (Cu), Gallium (Ga), Nickel (Ni), Chromium (Cr), and Indium (In) in the Xiang-Shan wetland’s surface sediments ranged from 23445.00 to 42123.33, 19234.50 to 51850.00, 266.05 to 764.73, 58.35 to 134.60, 60.20 to 252.05, 11.05 to 117.80, 57.35 to 121.63, 15.55 to 45.25, 46.90 to 112.87, and 17.90 to 56.23 mg.kg^−1^, respectively. While Lead (Pb) and Cadmium (Cd) were blow the detection limits at all studied stations in both seasons. The average contents of the 12 HMs in the Xiang-Shan wetland’s surface sediments showed a decreasing sequence of Al > Fe > Mn > Zn > Co > Ga > Cr > Cu > In > Ni > Pb = Cd. The ANOVA revealed discernible variances for Mn, Cu, Co, Cr, and In values across stations (*p* < 0.05).

**Figure 8:**
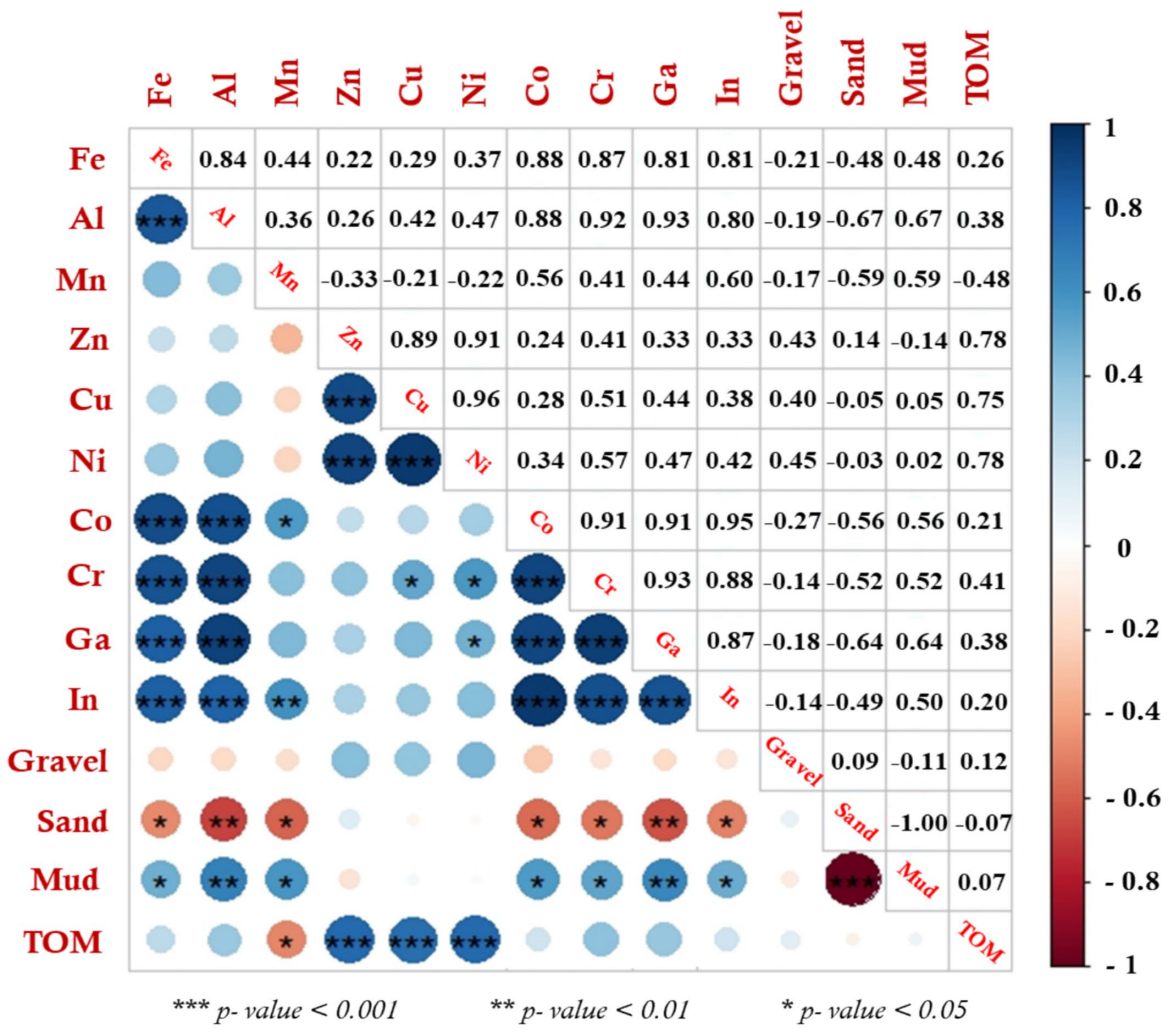
The association relationships among the analyzed parameters in Xiang-Shan wetlands’ sediments using Pearson’s correlation coefficient (KY: KeYa, KW: KeYa Water Supply Center, DJ: DaJuang, HM: HuiMin, FC: FongCin, HS: HaiShan, OB: Oyster Bed, YK: YenKan, MA: Mangrove Area).

Spatially, the greatest mean annual Fe, Al, Co, Cr, In, and Ga concentrations were observed in the DJ station, Zn, Cu, and Ni in the KY station, and Mn in the OB station. This may be attributed to the prevalence of fine-grained sediments with considerable amounts of OM in these stations, that have a tendency to bind with HMs. In addition to the existence of terrigenous freshwater sources and unprocessed domestic sewage from the surrounding area (25, 91, 92). Previously, Barik et al. (93) and Dar and El-Saharty (94) observed that fine-grained sediments have a higher affinity for metals owing to their large surface area and abundance of organic matter. Also, this observation was consistent with an earlier study by Tian et al. (8), who reported that fine sediments serve a critical role in controlling the mobility of HMs and subsequently their distributions in sediments. Furthermore, the Pearson’s correlation coefficient (Figure 8) revealed that most metals have high and significant positive associations with mud % and negative associations with sand % and gravel %, confirming the higher deposition and retention of metals by fine-grained sediments in the Xiang-Shan wetland. Conversely, in low-depth marine sediments, Giannico et al. (95, 96) investigated the concentrations and hazards of organic matters such as PCDD, PCDF, and PCBs. This study found high concentrations of dioxins and PCBs in marine sediments from Mar Piccolo 1^st^ Inlet, Italian Taranto, due to industrial settlements nearby, which are known potential sources of PCDD/Fs and PCBs (e.g., groundwater and freshwater pollution in the northern area of the basin).

Besides, the obtained results were compared to those previously presented in other investigations, as well as the average shale values (ASVs), to better comprehend the contamination status of HMs in sediments (Supplementary Table 3). The seasonal mean of Fe, Co, Ga, and In (30,464.07, 92.27, 91.44, and 36.61 mg.kg^−1^, respectively) was greater than those of most other areas around the globe, such as the Western Saronikos Gulf, Greece (97), the Dhaleshwari River in Bangladesh (98), the Gulf of Aqaba along the Saudi Arabia coastline (99), Bafa Lake in Turkey (38), the Xiang-Shan wetland in Taiwan (100), and the wetlands and main rivers in Taiwan (101). While the total average of Al (36690.77 mg.kg^−1^) was less than Changjiang River Estuary, China (102), and more than those of other earlier investigations. Likewise, the seasonal mean of Zn and Cu (116.52 and 38.21 mg.kg^−1^, respectively) was greater than those observed in other studies (Table 3) but lower than the upper levels of baseline concentrations in Taiwan (103). The value of Mn (553.50 mg.kg^−1^) was less than those of the Western Saronikos Gulf, Greece (97), but more than the levels in the Iranian Urmia Lake (90), and Bafa Lake in Turkey (38). Moreover, the mean annual Ni concentration (24.44 mg.kg^−1^) across all sampling stations was lower than those of most previous studies and higher than the shorelines of the Bohai and Yellow Seas in China (8), the Aqaba Gulf along the Saudi Arabia coastline (99), and the Western Taiwan Strait, China (104), but it was similar to the results of wetlands and main rivers in Taiwan (101). When compared with the average shale values (ASVs) that were established by Turekian and Wedepohl (56), the mean annual levels of all analyzed metals were below the ASVs, with the exception of Zn, Co, Ga, and In were comparable (Supplementary Table 3).

As a result of the spatial variability observed in the sediments, the overall concentration of HMs may not accurately reflect the current contamination levels. Hence, the HM concentrations alone are insufficient to assess the pollution level of HM in the sediments. Further quantitative indicators (e.g., E*_f_*, I*_geo_*, PLI, mC*_deg_*, P*_N_*, E*_R_*, and PERI) that consider the ASVs in the associated sediments are required.

### Assessment of heavy metals contamination

Single-element contamination indices, such as the enrichment factor (Ef) and geoaccumulation index (Igeo), were applied to assess the contamination of HM in the sediments (105). These indices provide information about how a particular metal is concentrated at a location of interest in comparison to the background.

Here, the mean E*_f_* values of Al, Co, Zn, Pb, Cu, Cr, Ni, Ga, In, Mn, and Cd were 0.70, 7.40, 1.99, 0.00, 1.35, 1.31, 0.58, 7.52, 554.85, 1.02, 0.00, respectively. These results revealed that the surface sediments of Xiang-Shan wetland were extremely enriched with In (E*_f_* > 50), heavily enriched with Co and Ga (5 ≤ E*_f_* < 10), and had nil to minor enrichment with the other heavy metals (1 ≤ E*_f_* < 3). The mean E*_f_* values of Al, Pb, and Cd were below one at all studied stations in both seasons, suggesting no enrichment and proving that they are largely originating from shale components or natural weathering activities. Conversely, the E*_f_* levels for Zn, Cr, Cu, Co, Ni, In, Mn, and Ga are almost more than one, implying a tendency from minor to extremely anthropogenic enrichment.

Regarding I*_geo_* index, the surface sediments of Xiang-Shan wetland were marked as nil or minor polluted with Fe, Cu, Al, Cd, Mn, Ni, Zn, Cr, and Pb (I*_geo_* < 1). Moreover, the average I*_geo_* values of Co, and Ga are categorized as moderately polluted in DJ, OB, and MA stations. The seasonal mean I*_geo_* values of Fe, Cu, Al, Cd, Mn, Ni, Zn, Cr, Ga, Pb, In, and Co were 0.13, 0.17, 0.09, 0.00, 0.13, 0.07, 0.25, 0.17, 0.94, 0.00, 70.54, and 0.94, respectively, indicating the range of uncontaminated (I*_geo_* < 1) to extremely polluted (I*_geo_* > 5).

The mean E*_f_* levels for the examined HMs were in the decreasing sequence of In > Ga > Co > Zn > Cu > Cr > Mn > Al > Ni > Pb = Cd, and the mean I*_geo_* declined in the following order: In > Ga ≥ Co > Zn > Cu ≥ Cr > Mn ≥ Fe > Al > Ni > Pb = Cd. As can be observed, the HMs have a similar order with regard to the estimated E*_f_* and I*_geo_*. Interestingly, E*_f_* and I*_geo_* values for Indium (In) metal at all sampling stations showed great values, suggesting extremely contamination; this is likely attributed to the industrial effluent from Hsinchu Science Industrial Park (HSIP). This park is the biggest industrial region in Taiwan, containing various high-tech companies producing photovoltaic plates, biomedical materials, liquid-crystal displayers (LCD), light-emitting diodes (LED), etc. (46). Gallium and Indium are crucial transition elements that are used in large quantities in the aforementioned industries, and they are discharged into the coastal zone of the study area via the KeYa river during the fabrication processes (i.e., cleaning operations, epitaxy, and chip fabrication in the production of high-speed semiconductors and LEDs), causing adverse impacts on humans (106–108).

To further determine the HM pollution in the surface sediments, the integrated pollution indices (PLI, mC*_deg_*, and P*_N_*) were used to estimate the overall HMs pollution in the Xiang-Shan wetland’s surface sediments. These indices were quantified from the contamination factor (*C_f_*) or pollution index (PI) of every single element. The pollution level identified by *C_f_* or PI in the Xiang-Shan wetland was comparable to the findings by E*_f_* and I*_geo_* described earlier. The average levels of *C_f_* or PI revealed a decreasing sequence of In (351.51) > Ga (4.70) > Co (4.68) > Zn (1.23) > Cu (0.86) > Cr (0.83) > Mn (0.64) > Fe (0.63) > Al (0.44) > Ni (0.36) > Pb = Cd (0.00). According to the classification of Chakraborty et al. (109) and Tian et al. (62) (Table 1), these data suggest that the surface sediments of Xiang-Shan wetland were highly contaminated with In (*C_f_* > 6), considerably polluted with Ga and Co (3 < *C_f_* ≤ 6), and unpolluted with the other metals (*C_f_* < 1).

Due to the great contribution of Indium (In), Gallium (Ga), and Cobalt (Co) metals in our study, the obtained data of PLI, mC*_deg_*, and P*_N_* displayed a certain level of HM contamination. The seasonal mean values of PLI ranged between 1.96 and 1.20, indicating the surface sediment of the investigated area were slightly polluted (1 ≤ PLI < 2). Specifically, the surface sediments of DJ, KY, and OB stations in the spring season are greater than 2, suggesting strong pollution with heavy metals, and the mean PLI showed the descending order of DJ (1.96) > KY (1.65) > OB (1.64) > MA (1.50) > HM (1.47) > HS (1.29) > FC (1.24) ≥ KW (1.24) > YK (1.20).

While the annual average mC*_deg_* values at all sampling stations fluctuated from 45.34 to 19.80, reflecting the range of severe (16 ≤ mC*_deg_* < 32) to extreme pollution (mC*_deg_* > 32). The mean value of mC*_deg_* was higher than 32 for DJ and OB stations in both seasons, reflecting extremely contamination in the sediments of these two stations while other stations’ sediments were heavily or severely polluted. The magnitude of mC*_deg_* levels between investigated stations was in the sequence of DJ (45.34) > OB (40.89) > MA (32.23) > HS (30.82) > HM (30.10) > KY (28.29) > FC (23.74) > KW (23.19) > YK (19.80).

Additionally, The Nemerow integrated pollution index (P*_N_*) is another widely employed proxy to quantify the pollution of HMs across all sampling stations. This index was calculated from the single pollution index (PI) of HMs mentioned previously. According to the mean P*_N_* values, the surface sediments of Xiang-Shan wetland were extremely polluted with In, Ga, and Co (class 4, P*_N_* > 3) in both two seasons and unpolluted (class 0, P*_N_* < 0.7) to significantly polluted (class 3, P*_N_* < 3) with the other metals (Table 2). The seasonal mean P*_N_* levels for the twelve HMs decreased as follows: In > Ga > Co > Zn > Cu > Cr > Fe ≥ Mn > Al > Ni > Pb = Cd.

### Evaluate the potential risks of metals to the environment

The possible hazards related to the examined elements in the Xiang-Shan wetland’s surface sediments were evaluated utilizing the potential ecological risk index (PERI) and consensus-based sediment quality guidelines (SQGs).

PERI demonstrates the risks posed by pollutants and shows the susceptibility of ecological communities to given metals (110). The average E*_R_* of HMs varied considerably. The E*_R_* levels for the eight elements were ordered descendingly as follows: Co (23.39) > Cu (4.29) > Ni (1.79) > Cr (1.65) > Zn (1.23) > Mn (0.64) for both spring and winter sediments. Accordingly, all HMs across all sampling stations exhibited a minimal risk to the ecology with E*_R_* levels below 30 (E*_R_* < 30). Specifically, the E*_R_* values for Co exceeded 30 at DJ (in both seasons) and OB (in the spring season) stations, indicating Co had moderate ecological risk in these two stations. Comprehensively, the seasonal mean PERI values of the surface sediments were 39.21, 24.38, 49.17, 32.59, 24.83, 28.22, 39.61, 23.91, and 35.02 in KY, KW, DJ, HM, FC, HS, OB, YK, and MA, respectively, with a total mean of 32.99. According to the PERI classifications, all sampling stations showed minimal ecological risk (PERI <40), with the exception of the OB station in the spring season and the DJ station in both seasons, which posed a moderate risk (40 ≤ PERI <80) to the environment, mostly due to Co contamination. Similar to mC*_deg_* and PLI, the average PERI levels of the spring sediments were greater than those of the winter season, and they showed the descending order of DJ (49.17) > OB (39.61) > KY (39.21) > MA (35.02) > HM (32.59) > HS (28.22) > FC (24.83) > KW (24.38) > YK (23.91).

Similarly, sediment quality guidelines (SQGs) are the most prevalent conventional approach for determining the likely adverse impacts of HMs in sediments (75, 111, 112). Generally, these guidelines have low and high limits for various heavy metals. Supplementary Table 4 juxtaposes our results with numerous SQGs’ reference values. The reference data imposed by the National Oceanic and Atmospheric Administration of the USA (NOAA) (72) are equivalent to those of the Australian and New Zealand Environment and Conservation Council and the Agriculture and Resource Management Council of Australia and New Zealand (71). Those developed by the Taiwan EPA (74) and the Canadian Council of Ministers of the Environment (73) are analogous to each other. Overall, the last two have somewhat lower values than the previous two, implying that the latter two reflect more rigorous values for the SQG technique. In comparison with the values of SQGs, the mean value of Cr greatly surpassed CCME’s ISQG, but it was close to the lower limit of Taiwan’s EPA. Similarly, the Cu value exceeded the lower limits of the CCME’s ISQG and NOAA’s effects range-low (ERL). Ni concentration in our work is between the lower and upper limits of NOAA’s ERL and ANZECC & ARMCANZ, but it is comparable to the Taiwan EPA’s lower limit. Meanwhile, the mean Zn value was considerably greater than the Taiwan EPA’s lower limit. Finally, the contents of Pb and Cd in our research were below all of the referenced levels established in the other guidelines. Overall, the mean levels of Zn, Cr, Ni, and Cu in the current research exceeded the lower limits of various SQGs, indicating that HM risk rarely occurs in the sediment of the Xiang-Shan wetland (105).

### Identify the potential sources of HMs in the Xiang-Shan wetland’s sediments

The assessment of current contamination alone is inadequate to reduce the level of HMs pollution in the Xiang-Shan wetland’s surface sediments. Various bivariate and multivariate statistical methods, including Pearson’s correlation coefficient (PCC), Hierarchical cluster analysis (HCA), and principle component analysis (PCA), have been shown to be useful for examining the correlations and identifying the possible sources of HMs in sediments (26, 36).

Regarding PCC, a positive correlation among two variables implies that they originate from common origins and similar migration ways, while a negative correlation reflects distinct origins and is likely related to lithogenic or natural activities (39). Statistically, the correlation coefficient (r) can be categorized into four levels: *r* < ± 0.5 negligible, 0.5 ± ≤ *r* < ± 0.6 significant, 0.6 ± ≤ *r* < ± 0.7 high, and *r* ≥ ± 0.7 strong. As shown in Figure 8, there were strong positive correlations (*r* ≥ 0.7, *p* < 0.001) among some studied variables, and the strongest associations, in decreasing order of correlation coefficient, were between the content of Cu-Ni (0.96), Co-In (0.95), Al-Ga (0.93), Cr-Ga (0.93), Al-Cr (0.92), Zn-Ni (0.91), Co-Cr (0.91), Co-Ga (0.91), Zn-Cu (0.89), Fe-Co (0.88), Al-Co (0.88), Cr-In (0.88), Fe-Cr (0.87), Ga-In (0.87), Fe-Al (0.84), Fe-Ga (0.81), Fe-In (0.81), Al-In (0.80), Zn-TOM (0.78), Ni-TOM (0.78), and Cu-TOM (0.75). In addition, Al-Mud (0.67), Ga-Mud (0.64), and Mn-In (0.60) showed high positive correlation (0.6 ≤ *r* < 0.7, *p* < 0.01), while Mn-Mud (0.59), Ni-Cr (0.57), Mn-Co (0.56), Co-Mud (0.56), Cr-Mud (0.52), Cu-Cr (0.51), and In-Mud (0.50) showed significant correlation (0.5 ≤ *r* < 0.6, *p* < 0.05). Contrarily, most metals had a negative and negligible relationship with sand and gravel, respectively. Similar findings were observed previously by Liang et al. (113) and Briki et al. (114), who confirmed that positive relationships between heavy metals imply similar anthropogenic pollution sources and migration processes, whereas negative correlations indicate that they originated from various sources, which are likely geogenic.

**Figure 9:**
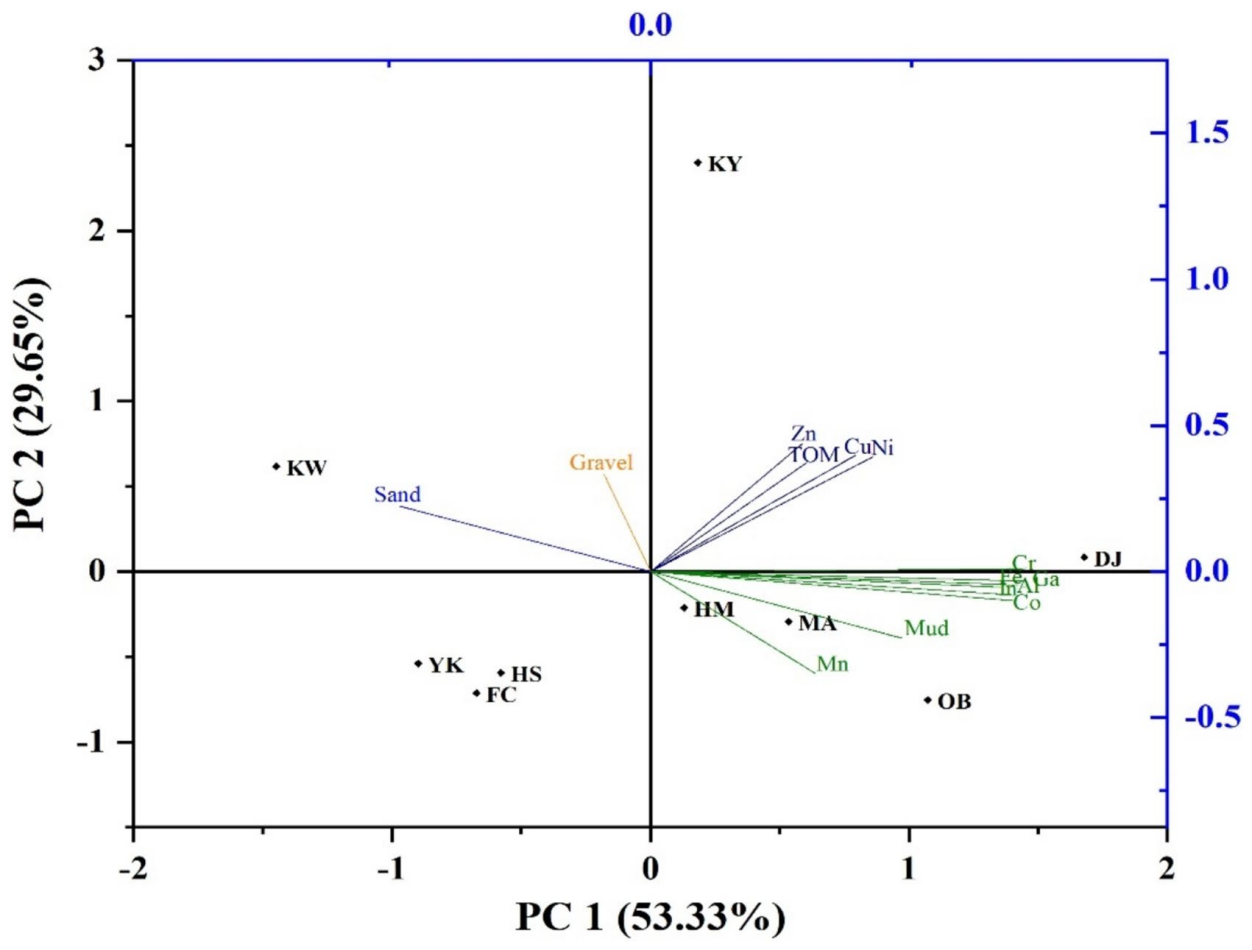
Biplot depicts the PCA analysis for HMs, TOM, and GSA in sediments of the Xiang-Shan wetland (KY: KeYa, KW: KeYa Water Supply Center, DJ: DaJuang, HM: HuiMin, FC: FongCin, HS: HaiShan, OB: Oyster Bed, YK: YenKan, MA: Mangrove Area).

PCA was performed to further investigate the association, HMs sources, and the linked interactions of HMs and sediment properties (i.e., TOM%, gravel%, sand%, and mud%). The PCA observations illustrated that the variance of HMs, TOM, and GSA can be described by two principal components that explained 82.98% of the cumulative variance. PC1 and PC2 explained 53.33 and 29.65%, respectively. As shown in Figure 9, Fe, Al, Co, Cr, Ga, In, Mn, and mud were positively associated with the first component (PC1), indicating that these variables predominantly came from similar sources, and PCC data confirm the possibility that these HMs had common origins. Inversely, sand and gravel variables were negatively loaded with PC1, indicating that the heavy metal distribution is highly affected by muddy sediments in DJ, OB, HM, and MA stations. Moreover, the PC2 had positively loaded Zn, Cu, Ni, and TOM, reflecting that these metals came from another source.

**Figure 10:**
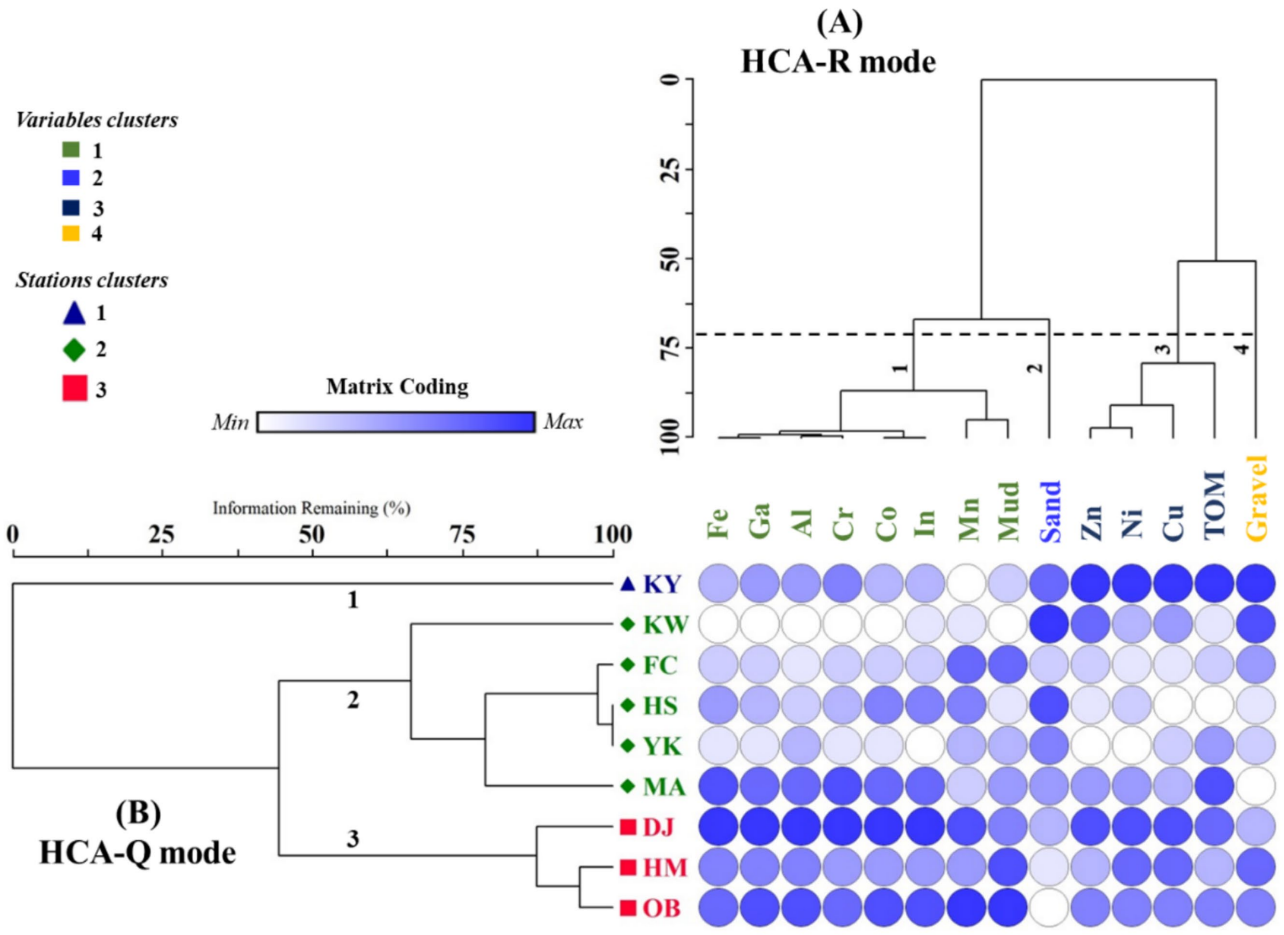
A dendrogram shows the hierarchical cluster analysis. **(A)** HCA for HMs, TOM, and GSA, and **(B)** HCA for studied stations (KY: KeYa, KW: KeYa Water Supply Center, DJ: DaJuang, HM: HuiMin, FC: FongCin, HS: HaiShan, OB: Oyster Bed, YK: YenKan, MA: Mangrove Area).

Similar to PCC and PCA, HCA (HCA-R mode) was conducted using the method of Euclidean distance to study similar heavy metal interrelationships and explore their potential origins (26). The HCA dendrogram provided data that split the PC1 and PC2 components into four distinct clusters with more precise similarities (Figure 10A). Cluster 1 contains Fe, Ga, Al, Cr, Co, In, Mn, and mud-grained size, proving that these metals emanated from a similar terrigenous source (115–117). Fe and Al elements are abundant in the crust of the earth and naturally enter aquatic environments, as well as serving a significant role in HMs scavenging and their incorporation into sediments (118). As a result, the presence of Fe and Al with Ga, Co, In, and Cr can suggest diversity in pollutant sources between natural and anthropogenic activities. Also, the strong positive correlation among Co, Ga, and In might be due to industrial effluent from the industrialized urban area. Furthermore, the significant positive relationship of mud with Mn, Al, Fe, Co, Cr, Ga, and In indicates that mud-grained particles can extensively influence the mobility of these seven metals (25, 119, 120). Cluster 3 consists of Zn, Ni, Cu, and TOM; this data implied that the TOM content may have an influence on the distribution of HMs in surface sediments owing to its strong affinity through adsorption or complexation (8, 121, 122). Our findings coincided with earlier observations by Liu et al. (123), who mentioned that the HM concentrations in the Luanhe Estuary sediments were influenced by the OM content. Additionally, cluster 2 and 4 comprise only sand and gravel, respectively; it seems that the gravel and sand sediments have a negligible influence on the HM distribution. Based on the heavy metal’s distribution (HCA-Q mode), the main nine studied stations were categorized into three clusters (Figure 10B). The first cluster contains one station (KY). This cluster had the greatest contents of Zn, Cu, Ni, and TOM. The second cluster comprises four stations (KW, FC, HS, YK, and MA), which had the highest percentage of sand (KW) and relative high values of TOM, Fe, Ga, Al, Cr, Co, and In (MA). While the third one contains three stations (DJ, HM, and OB), which had the greatest values of Fe, Al, Co, Cr, In, and Ga (DJ), Mn, and mud (OB). The results of HCA were consistent with the PCC and PCA data.

In summary, Fe and Al in cluster 1 enter the sediment of the wetland from another natural origin unrelated to organic matter. In contrast, In, Ga, Co, Cr, and Mn are primarily derived in the wetland sediment from anthropogenic origins, in addition to natural sources related to Fe and Al. Unlike cluster 1, heavy metals in cluster 3 (Zn, Cu, and Ni) are linked to OM and carried into the wetland while affixed to OM that derives mostly from natural origins. These outcomes support other findings and are reinforced by the sediment contamination indices discussed in this work. Restoration of Xiang-Shan wetland requires the local government to implement measures to prevent HM pollution as a matter of urgency, particularly in relation to In, Ga, Co, Cr, and Mn. In order to reveal the full ecological risk posed by these HMs, extensive ecotoxicological studies are required on the responses of the biota of Xiang-Shan wetland to these toxic metals.

## Conclusion

The Xiang-Shan wetland is a natural home for millions of crustaceans, prawns, benthic invertebrates, shellfish, and endangered avian species. Added to that, its economic value to the government of Hsinchu City. Thus, it is critical to evaluate the heavy metal contamination and identify its ecological threat. The average values of the 12 metals in the Xiang-Shan wetland’s surface sediments showed a decreasing sequence of Al > Fe > Mn > Zn > Co > Ga > Cr > Cu > In > Ni > Pb = Cd. The single pollution indices proved that the majority of sampling stations were unpolluted to minor polluted by Fe, Al, Zn, Cu, Mn, Cr, and Ni, moderately to heavily polluted by Co and Ga, and extremely polluted by In at all studied stations. The findings of PLI demonstrated that about 67% of spring sediments and entirely of winter sediments were moderately polluted (PLI < 2). Based on PERI, about 67% of spring sediment and 89% of winter sediment posed “minimal ecological risk” (PERI <40). Multivariate analyses demonstrated that Fe, Al, Zn, Cu, and Ni came from natural origins, while the sources of Co, Ga, In, Cr, and Mn were both anthropogenic and natural. Our research sounds the alarm for stricter management of metal discharges, and it is critical for the integrity of the ecosystem that heavy metals in aquatic-sedimentary systems in the Xiang-Shan wetland are continuously monitored.

## Data Availability

The raw data supporting the conclusions of this article will be made available by the authors, without undue reservation.

## References

[1] Castillo MA Trujillo IS Alonso EV de Torres AG Pavón JC. Bioavailability of heavy metals in water and sediments from a typical Mediterranean Bay (Málaga Bay, region of Andalucía, southern Spain). Mar Pollut Bull. (2013) 76:427–34. doi: 10.1016/j.marpolbul.2013.08.031, PMID: 24054786

[2] Gopinath A Nair S Kumar N Jayalakshmi K Pamalal D. A baseline study of trace metals in a coral reef sedimentary environment, Lakshadweep archipelago. Environ Earth Sci. (2010) 59:1245–66. doi: 10.1007/s12665-009-0113-6

[3] Nobi E Dilipan E Thangaradjou T Sivakumar K Kannan L. Geochemical and geo-statistical assessment of heavy metal concentration in the sediments of different coastal ecosystems of Andaman Islands, India. Coastal Shelf Sci. (2010) 87:253–64. doi: 10.1016/j.ecss.2009.12.019

[4] Ramachandra T Sudarshan P Mahesh M Vinay S. Spatial patterns of heavy metal accumulation in sediments and macrophytes of Bellandur wetland, Bangalore. J Environ Manag. (2018) 206:1204–10. doi: 10.1016/j.jenvman.2017.10.014, PMID: 29157887

[5] Rezapour S Atashpaz B Moghaddam SS Damalas CA. Heavy metal bioavailability and accumulation in winter wheat (*Triticum aestivum* L.) irrigated with treated wastewater in calcareous soils. Sci Total Environ. (2019) 656:261–9. doi: 10.1016/j.scitotenv.2018.11.288, PMID: 30504026

[6] Shu Q Ma Y Liu Q Zhang S Hu Z Yang P. Levels and ecological risk of heavy metals in the surface sediments of tidal flats along the North Jiangsu coast, China. Mar Pollut Bull. (2021) 170:112663. doi: 10.1016/j.marpolbul.2021.112663, PMID: 34218032

[7] Hosono T Su C-C Delinom R Umezawa Y Toyota T Kaneko S. Decline in heavy metal contamination in marine sediments in Jakarta Bay, Indonesia due to increasing environmental regulations. Estuar Coast Shelf Sci. (2011) 92:297–306. doi: 10.1016/j.ecss.2011.01.010

[8] Tian K Wu Q Liu P Hu W Huang B Shi B. Ecological risk assessment of heavy metals in sediments and water from the coastal areas of the Bohai Sea and the Yellow Sea. Environ Int. (2020) 136:105512. doi: 10.1016/j.envint.2020.105512, PMID: 31999973

[9] Fu F Wang QJ. Removal of heavy metal ions from wastewaters: a review. J Environ Manag. (2011) 92:407–18. doi: 10.1016/j.jenvman.2010.11.011, PMID: 21138785

[10] Barut IF Ergin M Meriç E Avşar N Nazik A Suner F. Contribution of natural and anthropogenic effects in the Iznik Lake bottom sediment: geochemical and microfauna assemblages evidence. Quat Int. (2018) 486:129–42. doi: 10.1016/j.quaint.2017.10.026

[11] Shajib MTI Hansen HCB Liang T Holm PE. Metals in surface specific urban runoff in Beijing. Environ Pollut. (2019) 248:584–98. doi: 10.1016/j.envpol.2019.02.039, PMID: 30836240

[12] Zahra A Hashmi MZ Malik RN Ahmed ZJ. Enrichment and geo-accumulation of heavy metals and risk assessment of sediments of the Kurang Nallah—feeding tributary of the Rawal Lake reservoir, Pakistan. Sci Total Environ. (2014) 470-471:925–33. doi: 10.1016/j.scitotenv.2013.10.017, PMID: 24239813

[13] Bastami KD Neyestani MR Molamohyedin N Shafeian E Haghparast S Shirzadi IA. Bioavailability, mobility, and origination of metals in sediments from Anzali wetland, Caspian Sea. Caspian Sea Marine Pollution Bulletin. (2018) 136:22–32. doi: 10.1016/j.marpolbul.2018.08.059, PMID: 30509802

[14] Xu X Cao Z Zhang Z Li R Hu B. Spatial distribution and pollution assessment of heavy metals in the surface sediments of the Bohai and yellow seas. Mar Pollut Bull. (2016) 110:596–602. doi: 10.1016/j.marpolbul.2016.05.079, PMID: 27269383

[15] Anbuselvan N Sridharan M. Heavy metal assessment in surface sediments off Coromandel Coast of India: implication on marine pollution. Mar Pollut Bull. (2018) 131:712–26. doi: 10.1016/j.marpolbul.2018.04.074 29886998

[16] Gholizadeh M Patimar R. Ecological risk assessment of heavy metals in surface sediments from the Gorgan Bay, Caspian Sea. Mar Pollut Bull. (2018) 137:662–7. doi: 10.1016/j.marpolbul.2018.11.009, PMID: 30503481

[17] Nour HES. Distribution, ecological risk, and source analysis of heavy metals in recent beach sediments of Sharm El-sheikh, Egypt. Environ Monit Assess. (2019) 191:546. doi: 10.1007/s10661-019-7728-1, PMID: 31392419

[18] Ye Z Chen J Gao L Liang Z Li S Li R. 210Pb dating to investigate the historical variations and identification of different sources of heavy metal pollution in sediments of the Pearl River estuary, southern China. Mar Pollut Bull. (2020) 150:110670. doi: 10.1016/j.marpolbul.2019.110670, PMID: 31669709

[19] Xu F Hu B Yuan S Zhao Y Dou Y Jiang Z. Heavy metals in surface sediments of the continental shelf of the South Yellow Sea and East China Sea: sources, distribution and contamination. Catena. (2018) 160:194–200. doi: 10.1016/j.catena.2017.09.022

[20] Şimşek A Özkoç HB Bakan G. Environmental, ecological and human health risk assessment of heavy metals in sediments at Samsun-Tekkeköy, north of Turkey. Environ Sci Pollut Res. (2022) 29:2009–23. doi: 10.1007/s11356-021-15746-w, PMID: 34363161

[21] Rezapour S Siavash Moghaddam S Nouri A Khosravi AK. Urbanization influences the distribution, enrichment, and ecological health risk of heavy metals in croplands. Sci Rep. (2022) 12:3868. doi: 10.1038/s41598-022-07789-x, PMID: 35264644 PMC8907202

[22] Adamo P Arienzo M Imperato M Naimo D Nardi G Stanzione D. Distribution and partition of heavy metals in surface and sub-surface sediments of Naples city port. Chemosphere. (2005) 61:800–9. doi: 10.1016/j.chemosphere.2005.04.001, PMID: 15893789

[23] Bahloul M Baati H Amdouni R Azri C. Assessment of heavy metals contamination and their potential toxicity in the surface sediments of Sfax solar Saltern, Tunisia. Environ Earth Sci. (2018) 77:1–22. doi: 10.1007/s12665-018-7227-7, PMID: 40103751

[24] Wang Q Chen Q Yan D Xin S. Distribution, ecological risk, and source analysis of heavy metals in sediments of Taizihe River, China. Environ Earth Sci. (2018) 77:1–14. doi: 10.1007/s12665-018-7750-6, PMID: 40103751

[25] Salah-Tantawy A Chang C-SG Liu M-Y Young S-S. Exploring the diversity and structural response of sediment-associated microbiota communities to environmental pollution at the siangshan wetland in Taiwan using environmental DNA metagenomic approach. Front Mar Sci. (2022) 9:990428. doi: 10.3389/fmars.2022.990428

[26] Nour HE Helal SA Wahab MA. Contamination and health risk assessment of heavy metals in beach sediments of Red Sea and Gulf of Aqaba, Egypt. Mar Pollut Bull. (2022) 177:113517. doi: 10.1016/j.marpolbul.2022.113517, PMID: 35299149

[27] Ghrefat HA Abu-Rukah Y Rosen MA. Application of geoaccumulation index and enrichment factor for assessing metal contamination in the sediments of Kafrain dam, Jordan. Environ Monit Assess. (2011) 178:95–109. doi: 10.1007/s10661-010-1675-1, PMID: 20839049

[28] Bednarova Z Kuta J Kohut L Machat J Klanova J Holoubek I. Spatial patterns and temporal changes of heavy metal distributions in river sediments in a region with multiple pollution sources. J Soils Sediments. (2013) 13:1257–69. doi: 10.1007/s11368-013-0706-2

[29] Nazeer S Hashmi MZ Malik RN. Heavy metals distribution, risk assessment and water quality characterization by water quality index of the river Soan, Pakistan. Ecol Indic. (2014) 43:262–70. doi: 10.1016/j.ecolind.2014.03.010

[30] Cheng Q Wang R Huang W Wang W Li X. Assessment of heavy metal contamination in the sediments from the Yellow River wetland National Nature Reserve (the Sanmenxia section), China. Environ Sci Pollut Res. (2015) 22:8586–93. doi: 10.1007/s11356-014-4041-y, PMID: 25561267

[31] Mamat Z Haximu S Zhang ZY Aji R. An ecological risk assessment of heavy metal contamination in the surface sediments of Bosten Lake, Northwest China. Environ Sci Pollut Res. (2016) 23:7255–65. doi: 10.1007/s11356-015-6020-3, PMID: 26769477

[32] Chai L Li H Yang Z Min X Liao Q Liu Y. Heavy metals and metalloids in the surface sediments of the Xiangjiang River, Hunan, China: distribution, contamination, and ecological risk assessment. Environ Sci Pollut Res. (2017) 24:874–85. doi: 10.1007/s11356-016-7872-x 27761857

[33] Liu R Jiang W Li F Pan Y Wang C Tian H. Occurrence, partition, and risk of seven heavy metals in sediments, seawater, and organisms from the eastern sea area of Shandong peninsula, Yellow Sea, China. J Environ Manag. (2021) 279:1117. doi: 10.1016/j.jenvman.2020.111771 33307318

[34] Al-Kahtany K Nour HE El-Sorogy AS Alharbi T. Ecological and health risk assessment of heavy metals contamination in mangrove sediments, Red Sea coast. Mar Pollut Bull. (2023) 192:115000. doi: 10.1016/j.marpolbul.2023.115000, PMID: 37210984

[35] Hossain MB Sultana J Pingki FH Nur A-AU Mia MS Bakar MA. Accumulation and contamination assessment of heavy metals in sediments of commercial aquaculture farms from a coastal area along the northern bay of Bengal. Front Environ Sci. (2023) 11:1148360. doi: 10.3389/fenvs.2023.1148360

[36] Jamshidi-Zanjani A Saeedi M. Metal pollution assessment and multivariate analysis in sediment of Anzali international wetland. Environ Earth Sci. (2013) 70:1791–808. doi: 10.1007/s12665-013-2267-5

[37] Tang W Shan B Zhang H Zhang W Zhao Y Ding Y. Heavy metal contamination in the surface sediments of representative limnetic ecosystems in eastern China. Sci Rep. (2014) 4:7152. doi: 10.1038/srep07152, PMID: 25412580 PMC4239569

[38] Algül F Beyhan M. Concentrations and sources of heavy metals in shallow sediments in Lake Bafa, Turkey. Sci Rep. (2020) 10:11782. doi: 10.1038/s41598-020-68833-2, PMID: 32678245 PMC7366620

[39] Zhang Q Ren F Xiong X Gao H Wang Y Sun W. Spatial distribution and contamination assessment of heavy metal pollution of sediments in coastal reclamation areas: a case study in Shenzhen Bay, China. Environ Sci Eur. (2021) 33:1–11. doi: 10.1186/s12302-021-00532-9, PMID: 40093350

[40] Du Laing G Rinklebe J Vandecasteele B Meers E Tack F. Trace metal behaviour in estuarine and riverine floodplain soils and sediments: a review. Sci Total Environ. (2009) 407:3972–85. doi: 10.1016/j.scitotenv.2008.07.025, PMID: 18786698

[41] Lin Y-C Chang-Chien G-P Chiang P-C Chen W-H Lin Y-C. Multivariate analysis of heavy metal contaminations in seawater and sediments from a heavily industrialized harbor in southern Taiwan. Mar Pollut Bull. (2013) 76:266–75. doi: 10.1016/j.marpolbul.2013.08.027, PMID: 24054783

[42] Young S Jiang H Syu R Huang S. The biodiversity of siangshan wetland. government-authorized project final report (in Chinese), vol. 210. Taiwan: Hsinchu City Government, National Hsinchu teacher’s College (2005).

[43] Young S. The study of polychaeta and sipuncula from hisinchu city costal wildlife sanctuary, habitats and species distribution on temporal and spatial scale. Government authorized project final report (in Chinese), vol. 169. Taiwan: Taiwan governemnt (2009).

[44] Chang S Chiu H-M Tu W. The silence of silicon lambs: speaking out health and environmental impacts within Taiwan's Hsinchu science-based industrial park. IEEE International Symposium on Electronics and the Environment, 2004. Conference Record. 2004; (2004). IEEE; 258–263. doi: 10.1109/ISEE.2004.1299726

[45] Tu W-L. Challenges of environmental governance in the face of IT industrial dominance: a study of Hsinchu science-based Industrial Park in Taiwan. Int J Environ Sustain Dev. (2005) 4:290–309. doi: 10.1504/IJESD.2005.007742

[46] Wei K-Y. Multivariate analyses of potentially toxic elements along an industrialized Urban River in northern Taiwan. J Environ Prot. (2021) 12:983–1000. doi: 10.4236/jep.2021.1211057

[47] Modak D Singh K Chandra H Ray P. Mobile and bound forms of trace metals in sediments of the lower Ganges. Water Res. (1992) 26:1541–8. doi: 10.1016/0043-1354(92)90075-F

[48] Lin J-G Chen S Su C. Assessment of sediment toxicity by metal speciation in different particle-size fractions of river sediment. Water Sci Technol. (2003) 47:233–41. doi: 10.2166/wst.2003.0694, PMID: 12793685

[49] Folk RL. Petrology of sedimentary rocks. Austin: Hemphill Publishing Company (1974). 182 p.

[50] Wakley A Black C. Determination of organic matter in the soil by chromic acid digesion. Soil Sci. (1934) 63:251–64.

[51] Element C. Method 3051A microwave assisted acid digestion of sediments, sludges, soils, and oils. Für Anal Chem. (2007) 111:362–6.

[52] Sakan SM Đorđević DS Manojlović DD Predrag PS. Assessment of heavy metal pollutants accumulation in the Tisza river sediments. J Environ Manag. (2009) 90:3382–90. doi: 10.1016/j.jenvman.2009.05.013, PMID: 19515481

[53] Daskalakis KD O'Connor TP. Normalization and elemental sediment contamination in the coastal United States. Environ Sci Technol. (1995) 29:470–7. doi: 10.1021/es00002a024 22201394

[54] Zhang L Ye X Feng H Jing Y Ouyang T Yu X. Heavy metal contamination in western Xiamen Bay sediments and its vicinity, China. Marine Poll Bullet. (2007) 54:974–82. doi: 10.1016/j.marpolbul.2007.02.010, PMID: 17433373

[55] Ergin M Saydam C Baştürk Ö Erdem E Yörük R. Heavy metal concentrations in surface sediments from the two coastal inlets (Golden Horn estuary and Izmit Bay) of the northeastern sea of Marmara. Chem Geol. (1991) 91:269–85. doi: 10.1016/0009-2541(91)90004-B

[56] Turekian KK Wedepohl KH. Distribution of the elements in some major units of the earth's crust. Geological Society America Bullet. (1961) 72:175–92. doi: 10.1130/0016-7606(1961)72[175:DOTEIS]2.0.CO;2

[57] Acevedo-Figueroa D Jiménez B Rodríguez-Sierra C. Trace metals in sediments of two estuarine lagoons from Puerto Rico. Environ Pollut. (2006) 141:336–42. doi: 10.1016/j.envpol.2005.08.037, PMID: 16249046

[58] Muller G. Index of geoaccumulation in sediments of the Rhine River. Geo J. (1969) 2:108–18.

[59] Chen C-W Kao C-M Chen C-F Dong C-D. Distribution and accumulation of heavy metals in the sediments of Kaohsiung Harbor, Taiwan. Chemosphere. (2007) 66:1431–40. doi: 10.1016/j.chemosphere.2006.09.030, PMID: 17113128

[60] Zhang W Feng H Chang J Qu J Xie H Yu L. Heavy metal contamination in surface sediments of Yangtze River intertidal zone: an assessment from different indexes. Environ Pollut. (2009) 157:1533–43. doi: 10.1016/j.envpol.2009.01.007, PMID: 19217701

[61] Tomlinson D Wilson J Harris C Jeffrey D. Problems in the assessment of heavy-metal levels in estuaries and the formation of a pollution index. Helgoländer meeresuntersuchungen. (1980) 33:566–75. doi: 10.1007/BF02414780

[62] Tian K Huang B Xing Z Hu W. Geochemical baseline establishment and ecological risk evaluation of heavy metals in greenhouse soils from Dongtai, China. Ecol Indic. (2017) 72:510–20. doi: 10.1016/j.ecolind.2016.08.037

[63] Abrahim G Parker R. Assessment of heavy metal enrichment factors and the degree of contamination in marine sediments from Tamaki estuary, Auckland, New Zealand. Environ Monit Assess. (2008) 136:227–38. doi: 10.1007/s10661-007-9678-2, PMID: 17370131

[64] Nemerow NL. Stream, lake, estuary, and ocean pollution. (1991). Available at: https://www.osti.gov/biblio/7030475

[65] Yang Z Lu W Long Y Liu X. Prediction and precaution of heavy metal pollution trend in urban soils of Changchun City, urban environ. Urban Ecol. (2010) 23:1–4.

[66] Hakanson L. An ecological risk index for aquatic pollution control. Sedimentological Approach Water Research. (1980) 14:975–1001. doi: 10.1016/0043-1354(80)90143-8, PMID: 39919639

[67] Nabholz J. Environmental hazard and risk assessment under the United States toxic substances control act. Sci Total Environ. (1991) 109-110:649–65. PMID: 1815379 10.1016/0048-9697(91)90218-4

[68] Singh A Sharma RK Agrawal M Marshall F. Health risk assessment of heavy metals via dietary intake of foodstuffs from the wastewater irrigated site of a dry tropical area of India. Food Chem Toxicol. (2010) 48:611–9. doi: 10.1016/j.fct.2009.11.041, PMID: 19941927

[69] Douay F Pelfrêne A Planque J Fourrier H Richard A Roussel H. Assessment of potential health risk for inhabitants living near a former lead smelter. Part 1: metal concentrations in soils, agricultural crops, and homegrown vegetables. Environ Monit Assess. (2013) 185:3665–80. doi: 10.1007/s10661-012-2818-3, PMID: 22886627

[70] Li R Pan C Xu J Ding G Zou Y. Application of potential ecological risk assessment model based on Monte Carlo simulation. Res Environ Sci. (2012) 25:1336–43. Available at: http://www.hjkxyj.org.cn

[71] Anzecc A. Australian and New Zealand guidelines for fresh and marine water quality. Australian and New Zealand Environment and Conservation Council and Agriculture and Resource Management Council of Australia and New Zealand, Canberra. (2000) 1:1–314

[72] Long ER Macdonald DD Smith SL Calder FD. Incidence of adverse biological effects within ranges of chemical concentrations in marine and estuarine sediments. Environ Manag. (1995) 19:81–97. doi: 10.1007/BF02472006

[73] CCME. Canadian environmental quality guidelines, vol. 2 Canadian Council of Ministers of the Environment (2002). Available at: https://ccme.ca/en

[74] Taiwan EPA. Soil and groundwater pollution remediation act. Taipei, Taiwan: Taiwan Environmental Protection Administration (2010).

[75] Lin Q Liu E Zhang E Li K Shen J. Spatial distribution, contamination and ecological risk assessment of heavy metals in surface sediments of Erhai Lake, a large eutrophic plateau lake in Southwest China. Catena. (2016) 145:193–203. doi: 10.1016/j.catena.2016.06.003

[76] Patman P Denny L Churchill B. Using SPSS for descriptive statistical analysis. Oxford University Press: University of Tasmania (2013).

[77] Arkkelin D. Using SPSS to understand research and data analysis. Psychol Curricul Mat. (2014) 1:1–194. Available at: https://scholar.valpo.edu/psych_oer/1

[78] Varol M. Assessment of heavy metal contamination in sediments of the Tigris River (Turkey) using pollution indices and multivariate statistical techniques. J Hazard Mater. (2011) 195:355–64. doi: 10.1016/j.jhazmat.2011.08.051, PMID: 21890271

[79] Wei T Simko V Levy M Xie Y Jin Y Zemla J. Package “corrplot”: Visualization of a correlation matrix. R Foundation for Statistical Computing: Vienna, Austria (2017).

[80] Team RD. R: A language and environment for statistical computing. Copenhagen Business School: R Foundation for Statistical Computing (2010).

[81] Grandin U. PC-ORD version 5: a user-friendly toolbox for ecologists. J Veg Sci. (2006) 17:843–4. doi: 10.1111/j.1654-1103.2006.tb02508.x, PMID: 40103450

[82] Rea DK Hovan SA. Grain size distribution and depositional processes of the mineral component of abyssal sediments: lessons from the North Pacific. Paleoceanography. (1995) 10:251–8. doi: 10.1029/94PA03355

[83] Pontoni L La Vecchia C Boguta P Sirakov M D’Aniello E Fabbricino M. Natural organic matter controls metal speciation and toxicity for marine organisms: a review. Environ Chem Lett. (2022) 20:797–812. doi: 10.1007/s10311-021-01310-y

[84] Odum WE Heald EJ. The detritus-based food web of an. Estuar Res Chem Biol Estuar Syst. (1975) 1:265.

[85] Huc A. Origin and formation of organic matter in recent sediments and its relation to kerogen. Kerogen. (1980)

[86] Suresh G Sutharsan P Ramasamy V Venkatachalapathy R. Assessment of spatial distribution and potential ecological risk of the heavy metals in relation to granulometric contents of Veeranam lake sediments. India Ecotoxicol Environ Safety. (2012) 84:117–24. doi: 10.1016/j.ecoenv.2012.06.027, PMID: 22835728

[87] Taweel A Shuhaimi-Othman M Ahmad A. Assessment of heavy metals in tilapia fish (*Oreochromis niloticus*) from the Langat River and engineering Lake in Bangi, Malaysia, and evaluation of the health risk from tilapia consumption. Ecotoxicol Environ Saf. (2013) 93:45–51. doi: 10.1016/j.ecoenv.2013.03.031, PMID: 23642778

[88] Li H Davis A. Heavy metal capture and accumulation in bioretention media. Environ Sci Technol. (2008) 42:5247–53. doi: 10.1021/es702681j, PMID: 18754376

[89] Tang W Ao L Zhang H Shan B. Accumulation and risk of heavy metals in relation to agricultural intensification in the river sediments of agricultural regions. Environ Earth Sci. (2014) 71:3945–51. doi: 10.1007/s12665-013-2779-z

[90] Rezapour S Asadzadeh F Nouri A Khodaverdiloo H Heidari M. Distribution, source apportionment, and risk analysis of heavy metals in river sediments of the Urmia Lake basin. Sci Rep. (2022) 12:17455. doi: 10.1038/s41598-022-21752-w, PMID: 36261490 PMC9582006

[91] Yu T Zhang Y Zhang Y. Distribution and bioavailability of heavy metals in different particle-size fractions of sediments in Taihu Lake, China. Chem Spec Bioavailab. (2012) 24:205–15. doi: 10.3184/095422912X13488240379124

[92] El-Metwally ME Madkour AG Fouad RR Mohamedein LI Eldine HAN Dar MA. Assessment the leachable heavy metals and ecological risk in the surface sediments inside the Red Sea ports of Egypt. International. J Mar Sci. (2017):7. doi: 10.5376/ijms.2017.07.0023

[93] Barik SS Prusty P Singh RK Tripathy S Farooq S Sharma K. Seasonal and spatial variations in elemental distributions in surface sediments of Chilika Lake in response to change in salinity and grain size distribution. Environ Earth Sci. (2020) 79:1–18. doi: 10.1007/s12665-020-09009-z, PMID: 40103751

[94] Dar MA El-Saharty AA. Recycling and retention of some trace metals in the mangrove sediments, Red Sea, Egypt. Egypt J Aquat Res. (2006) 32:34–47.

[95] Giannico OV Baldacci S Desiante F Basile FC Franco E Fragnelli GR. PCDD/fs and PCBs in *Mytilus galloprovincialis* from a contaminated area in Italy: the role of mussel size, temperature and meteorological factors. Food Additives Contaminants. (2022) 39:1123–35. doi: 10.1080/19440049.2022.2059108, PMID: 35389328

[96] Giannico OV Desiante F Basile FC Franco E Baldacci S Fragnelli GR. Dioxins and PCBs contamination in mussels from Taranto (Ionian Sea, southern Italy): a seven years spatio-temporal monitoring study. Ann Ist Super Sanita. (2020) 56:452–61. doi: 10.4415/ANN_20_04_07, PMID: 33346171

[97] Filippi G Dassenakis M Paraskevopoulou V Lazogiannis K. Sediment quality assessment in an industrialized Greek coastal marine area (western Saronikos gulf). Biogeosciences. (2023) 20:163–89. doi: 10.5194/bg-20-163-2023

[98] Islam MAS Hossain ME Nahar K Majed N. Assessment of environmental Hazard and heavy metal contamination in Dhaleshwari River sediment: a toxicity based study on pollution. Pollution. (2023) 9:67–83. doi: 10.22059/poll.2022.342243.1455

[99] El-Sorogy AS Youssef M Al-Kahtany K Saleh M. Distribution, source, contamination, and ecological risk status of heavy metals in the Red Sea-Gulf of Aqaba coastal sediments, Saudi Arabia. Mar Pollut Bull. (2020) 158:111411. doi: 10.1016/j.marpolbul.2020.111411 32753195

[100] Young SS. The ecological and water quality monitoring on siangshan wetland (national scale) 2018–2019. Government-authorized project final report (in Chinese), vol. 204. Taiwan: Hsinchu City Government, National Tsing Hua University (2019).

[101] Ye YT Young SS. An investigation of the heavy metals distribution of the main rivers and wetlands in Hsinchu [dissertation/master thesis in Chinese]. Taiwan: National Tsing Hua University. (2008).

[102] Wang Q Huang X Zhang Y. Heavy metals and their ecological risk assessment in surface sediments of the Changjiang River estuary and contiguous East China Sea. Sustain For. (2023) 15:4323. doi: 10.3390/su15054323

[103] Lai YJ Chang PC Lee YT Hung YH Huang YH Chen SD. Establishment and discussion of soil heavy metal background concentrations in Taiwan. J Soil Groundwater Remediation. (2018) 5:143–62. doi: 10.6499/JSGR.201807_5(3).0003

[104] Zhai B Liu Z Wang X Bai F Wang L Chen Z. Assessment of heavy metal contamination in surface sediments in the western Taiwan Strait. Mar Pollut Bull. (2020) 159:111492. doi: 10.1016/j.marpolbul.2020.111492, PMID: 32892924

[105] Sarkar SK Sarkar SK. Geochemical speciation and risk assessment of trace metals in sediments of sundarban wetland. Ecotoxicol Relevance Remedial Measures. (2018) 10:145–72. doi: 10.1007/978-981-10-2793-2_6, PMID: 40099217

[106] Bu-Olayan A Thomas B. Bourgeoning impact of the technology critical elements in the marine environment. Environ Pollut. (2020) 265:115064. doi: 10.1016/j.envpol.2020.115064, PMID: 32806423

[107] Yang J-L Chen L-H. Toxicity of antimony, gallium, and indium toward a teleost model and a native fish species of semiconductor manufacturing districts of Taiwan. J Elem. (2018) 23:191–199. doi: 10.5601/jelem.2017.22.3.1470

[108] Nguyen CH Field JA Sierra-Alvarez RJ. Microbial toxicity of gallium-and indium-based oxide and arsenide nanoparticles. J Environ Sci Health A. (2020) 55:168–78. doi: 10.1080/10934529.2019.1676065, PMID: 31607225

[109] Chakraborty P Ramteke D Chakraborty S Nath BN. Changes in metal contamination levels in estuarine sediments around India–an assessment. Mar Pollut Bull. (2014) 78:15–25. doi: 10.1016/j.marpolbul.2013.09.044, PMID: 24211100

[110] Fu J Zhao C Luo Y Liu C Kyzas GZ Luo Y. Heavy metals in surface sediments of the Jialu River, China: their relations to environmental factors. J Hazard Mater. (2014) 270:102–9. doi: 10.1016/j.jhazmat.2014.01.044, PMID: 24561322

[111] El Nemr A El-Said GF Ragab S Khaled A El-Sikaily A. The distribution, contamination and risk assessment of heavy metals in sediment and shellfish from the Red Sea coast, Egypt. Chemosphere. (2016) 165:369–80. doi: 10.1016/j.chemosphere.2016.09.048, PMID: 27668715

[112] Duodu GO Ogogo KN Mummullage S Harden F Goonetilleke A Ayoko GA. Source apportionment and risk assessment of PAHs in Brisbane River sediment, Australia. Ecol Indic. (2017) 73:784–99. doi: 10.1016/j.ecolind.2016.10.038

[113] Liang A Wang Y Guo H Bo L Zhang S Bai Y. Assessment of pollution and identification of sources of heavy metals in the sediments of Changshou Lake in a branch of the three gorges reservoir. PLoS One. (2015) 22:16067–76. doi: 10.1007/s11356-015-4825-8, PMID: 26062470

[114] Briki M Zhu Y Gao Y Shao M Ding H Ji H. Distribution and health risk assessment to heavy metals near smelting and mining areas of Hezhang, China. Environ Monit Assess. (2017) 189:1–19. doi: 10.1007/s10661-017-6153-6, PMID: 28823066

[115] Li F Huang J Zeng G Yuan X Li X Liang J. Spatial risk assessment and sources identification of heavy metals in surface sediments from the Dongting Lake, middle China. J Geochem Explor. (2013) 132:75–83. doi: 10.1016/j.gexplo.2013.05.007

[116] Zhang P Qin C Hong X Kang G Qin M Yang D. Risk assessment and source analysis of soil heavy metal pollution from lower reaches of Yellow River irrigation in China. Sci Total Environ. (2018) 633:1136–47. doi: 10.1016/j.scitotenv.2018.03.228, PMID: 29758865

[117] Nour HES Ramadan F Aita S Zahran H. Assessment of sediment quality of the Qalubiya drain and adjoining soils, eastern Nile Delta, Egypt. Arab J Geosci. (2021) 14:1–13. doi: 10.1007/s12517-021-06891-0, PMID: 40103751

[118] Jagus A Khak V Rzetala MA Rzetala M. Accumulation of heavy metals in the bottom sediments of the Irkutsk reservoir. Int J Environ Health. (2013) 6:350–62. doi: 10.1504/IJENVH.2013.056976

[119] Gao X Li P. Concentration and fractionation of trace metals in surface sediments of intertidal Bohai Bay, China. Mar Pollut Bull. (2012) 64:1529–36. doi: 10.1016/j.marpolbul.2012.04.026, PMID: 22704147

[120] Kim S Yang DS Kim YS. Distribution of metal contamination and grain size in the sediments of Nakdong River, Korea. Environ Monit Assess. (2020) 192:502. doi: 10.1007/s10661-020-08475-z, PMID: 32648138 PMC7347678

[121] Jiang X Teng A Xu W Liu X. Distribution and pollution assessment of heavy metals in surface sediments in the Yellow Sea. Mar Pollut Bull. (2014) 83:366–75. doi: 10.1016/j.marpolbul.2014.03.020, PMID: 24703395

[122] Li H Kang X Li X Li Q Song J Jiao N. Heavy metals in surface sediments along the Weihai coast, China: distribution, sources and contamination assessment. Mar Pollut Bull. (2017) 115:551–8. doi: 10.1016/j.marpolbul.2016.12.039, PMID: 28007385

[123] Liu J Yin P Chen B Gao F Song H Li M. Distribution and contamination assessment of heavy metals in surface sediments of the Luanhe River estuary, northwest of the Bohai Sea. Mar Pollut Bull. (2016) 109:633–9. doi: 10.1016/j.marpolbul.2016.05.020, PMID: 27197763

[124] Förstner U Ahlf W Calmano W Kersten M. Sediment criteria development: Contributions from environmental geochemistry to water quality management. Sediments and environmental geochemistry: Selected aspects and case histories. Springer, Berlin, Heidelberg. (1990) 311–338.

