# Supplementary material for: Multivariate analyses to evaluate the contamination, ecological risk, and source apportionment of heavy metals in the surface sediments of Xiang-Shan wetland, Taiwan
Source: Front Public Health. 2025 Apr 9;13:1459060. doi: 10.3389/fpubh.2025.1459060 (PMC12014647; doi:10.3389/fpubh.2025.1459060)
Supplement: Supplementary file 1 [file Data_Sheet_1.PDF]

Supplementary Table 1: The average levels of heavy metal (mg.kg^{-1}, dry wt.) in the Xiang-Shan wetland's surface sediments in the spring and winter seasons.

Season	Station	location (n)	Heavy metals concentrations (mg.kg^{-1})											
			**Zn**	**Al**	**Ni**	**Fe**	**Cu**	**Mn**	**Co**	**Cr**	**In**	**Cd**	**Ga**	**Pb**
Spring	KY	n = 2	252.05	35400.00	45.25	27380.00	117.80	319.15	78.70	81.50	34.50	ND	90.95	ND
	KW	n = 2	133.20	19234.50	19.20	24325.00	31.60	434.35	69.25	55.35	28.15	ND	64.05	ND
	DJ	n = 3	184.30	51850.00	35.07	39810.00	86.63	759.93	134.60	112.87	56.23	ND	121.63	ND
	HM	n = 2	105.20	38435.00	23.05	29490.00	35.00	629.60	94.00	76.80	38.45	ND	98.85	ND
	FC	n = 2	82.65	25770.00	15.60	25705.00	16.85	619.55	83.35	59.90	30.95	ND	82.75	ND
	HS	n = 3	84.63	29173.33	18.53	28513.33	12.17	638.83	90.53	67.60	38.80	ND	90.07	ND
	OB	n = 3	115.30	45763.33	26.53	34513.33	36.33	764.73	121.53	93.87	48.77	ND	109.80	ND
	YK	n = 2	65.90	31906.50	15.55	24115.00	17.05	491.75	74.45	63.60	27.40	ND	84.45	ND
	MA	n = 3	104.77	45036.67	23.50	42123.33	28.33	434.13	108.67	91.23	38.20	ND	105.00	ND
Winter	KY	n = 2	233.80	34600.00	37.05	27185.00	77.15	266.05	79.20	69.45	30.10	ND	91.55	ND
	KW	n = 2	107.15	20785.00	22.90	23445.00	25.05	405.15	58.35	46.90	25.40	ND	57.35	ND
	DJ	n = 3	147.43	48285.00	33.27	38624.67	54.73	648.17	121.97	109.23	48.57	ND	106.40	ND
	HM	n = 2	80.85	37780.00	27.25	29335.00	62.15	575.90	73.30	71.10	30.90	ND	89.65	ND
	FC	n = 2	78.75	26280.00	17.55	25366.00	11.05	653.45	61.75	63.30	23.70	ND	75.65	ND
	HS	n = 3	73.17	29570.00	18.73	28036.67	11.37	586.00	80.90	61.93	32.63	ND	76.30	ND
	OB	n = 3	111.90	44101.00	21.20	33686.67	32.77	667.37	107.10	76.67	46.00	ND	96.40	ND
	YK	n = 2	60.20	31592.00	16.90	25295.50	12.15	421.10	66.85	53.60	17.90	ND	68.10	ND
	MA	n = 3	87.63	43163.33	21.43	27070.67	27.47	408.13	95.13	83.83	36.07	ND	99.90	ND
	Max.		252.05	51850.00	45.25	42123.33	117.80	764.73	134.60	112.87	56.23	ND	121.63	ND
	Min.		60.20	19234.50	15.55	23445.00	11.05	266.05	58.35	46.90	17.90	ND	57.35	ND
	Average		116.52	36690.77	24.44	30464.07	38.21	553.50	92.27	76.70	36.61	ND	91.44	ND
	p- value		0.057	0.135	0.069	0.122	***0.004***	***0.004***	***0.016***	***0.041***	***0.02***	-	0.122	-

Bold and Italic numbers indicate significant variance in metals across the stations ($p < 0.05$).
ND refers not detected.

Supplementary Table 2: The values of Nemerow integrated pollution (P_N) for each studied metal in the Xiang-Shan wetlands' sediments.

Station	PI for each heavy metal																							
	Zn		Al		Ni		Fe		Cu		Mn		Co		Cr		In		Cd		Ga		Pb	
	Spring	*Winter*	*Spring*	*Winter*	*Spring*	*Winter*	*Spring*	*Winter*	*Spring*	*Winter*	*Spring*	*Winter*	*Spring*	*Winter*	*Spring*	*Winter*	*Spring*	*Winter*	*Spring*	*Winter*	*Spring*	*Winter*	*Spring*	*Winter*
KY	2.65	2.46	0.44	0.43	0.67	0.54	0.58	0.58	2.62	1.71	0.38	0.31	4.14	4.17	0.91	0.77	345.00	301.00	0	0	4.79	4.82	0	0
KW	1.40	1.13	0.24	0.26	0.28	0.34	0.52	0.50	0.70	0.56	0.51	0.48	3.64	3.07	0.62	0.52	281.50	254.00	0	0	3.37	3.02	0	0
DJ	1.94	1.55	0.65	0.60	0.52	0.49	0.84	0.82	1.93	1.22	0.89	0.76	7.08	6.42	1.25	1.21	562.33	485.67	0	0	6.40	5.60	0	0
HM	1.11	0.85	0.48	0.47	0.34	0.40	0.62	0.62	0.78	1.38	0.74	0.68	4.95	3.86	0.85	0.79	384.50	309.00	0	0	5.20	4.72	0	0
FC	0.87	0.83	0.32	0.33	0.23	0.26	0.54	0.54	0.37	0.25	0.73	0.77	4.39	3.25	0.67	0.70	309.50	237.00	0	0	4.36	3.98	0	0
HS	0.89	0.77	0.36	0.37	0.27	0.28	0.60	0.59	0.27	0.25	0.75	0.69	4.76	4.26	0.75	0.69	388.00	326.33	0	0	4.74	4.02	0	0
OB	1.21	1.18	0.57	0.55	0.39	0.31	0.73	0.71	0.81	0.73	0.90	0.79	6.40	5.64	1.04	0.85	487.67	460.00	0	0	5.78	5.07	0	0
YK	0.69	0.63	0.40	0.39	0.23	0.25	0.51	0.54	0.38	0.27	0.58	0.50	3.92	3.52	0.71	0.60	274.00	179.00	0	0	4.44	3.58	0	0
MA	1.10	0.92	0.56	0.54	0.35	0.32	0.89	0.57	0.63	0.61	0.51	0.48	5.72	5.01	1.01	0.93	382.00	360.67	0	0	5.53	5.26	0	0
**$PI_{aver.}$**	**1.32**	**1.15**	**0.45**	**0.44**	**0.36**	**0.35**	**0.65**	**0.61**	**0.94**	**0.78**	**0.67**	**0.61**	**5.00**	**4.35**	**0.87**	**0.79**	**379.39**	**323.63**	**0**	**0**	**4.96**	**4.45**	**0**	**0**
**$PI_{max.}$**	**2.65**	**2.46**	**0.65**	**0.60**	**0.67**	**0.54**	**0.89**	**0.82**	**2.62**	**1.71**	**0.90**	**0.79**	**7.08**	**6.42**	**1.25**	**1.21**	**562.33**	**485.67**	**0**	**0**	**6.40**	**5.60**	**0**	**0**
**P_N**	**2.10** ^{d}	**1.92** ^{c}	**0.56** ^{a}	**0.53** ^{a}	**0.54** ^{a}	**0.46** ^{a}	**0.78** ^{b}	**0.72** ^{b}	**1.97** ^{c}	**1.33** ^{c}	**0.79** ^{b}	**0.70** ^{a}	**6.13** ^{e}	**5.48** ^{e}	**1.08** ^{c}	**1.02** ^{c}	**479.66** ^{e}	**412.68** ^{e}	**0** ^{a}	**0** ^{a}	**5.72** ^{e}	**5.06** ^{e}	**0** ^{a}	**0** ^{a}

a, b, c, d and e letters refer to unpolluted, minor, moderate, significant, and extremely pollution, respectively (**65**).

Supplementary Table 3: The average contents of HMs in the Xiang-Shan wetlands' sediments and comparison with those of other region and average shale values (ASVs).

Study sites	HM concentrations (Average)												Reference
	Zn	Al	Ni	Fe	Cu	Mn	Co	Cr	In	Cd	Ga	Pb	
**Xiang-Shan wetland, Hsinchu, Taiwan**	**116.52**	**36,690.77**	**24.44**	**30,464.07**	**38.21**	**553.50**	**92.27**	**76.70**	**36.61**	**ND**	**91.44**	**ND**	**Current study**
Western Saronikos Gulf, Greece	69.26	32,925.25	287.50	24,091.38	28.39	1003.25	--	257.63	--	--	--	37.31	**(95)**
Changjiang River Estuary, China	68.67	83,990.00	29.32	--	24.47	--	18.77	69.22	--	0.48	--	16.09	**(100)**
Dhaleshwari River, Bangladesh	--	--	170.62	13,645.80	--	--	--	169.20	--	2.56	--	218.36	**(96)**
Urmia Lake, Iran	--	--	60.50	--	20.25	55.80	--	--	--	1.40	--	19.86	**(90)**
Eastern sea area of Shandong Peninsula, China	26.07	--	--	--	19.52	--	--	22.61	--	0.15	--	15.47	**(33)**
Shorelines of Bohai and Yellow seas, China	77.98	--	23.70	--	--	--	--	56.80	--	0.16	--	26.79	**(8)**
Aqaba Gulf, Red sea, Saudi Arabia	24.00	--	14.00	3374.00	30.00	184.00	4.50	39.00	--	0.91	--	6.60	**(97)**
Lake Bafa, Turkey	33.72	--	155.53	26,060.00	22.64	439.20	15.08	71.38	--	0.66	--	12.18	**(38)**
Western Taiwan Strait, China	51.70	--	16.50	--	10.20	--	8.00	38.70	--	--	--	18.30	**(102)**
Xiang-Shan wetland, Hsinchu city, Taiwan	64.17	8669.91	34.64	18,878.26	25.02	--	--	68.64	1.32	0.82	36.14	39.97	**(98)**
Love River, Kaohsiung city, Taiwan	172.00	--	22.70	39.18	55.10	--	--	47.57	--	0.52	--	17.60	**(126)**
Wetlands and main rivers in Hsinchu city, Taiwan	112.32	12,173.95	24.88	--	36.92	--	--	22.33	10.05	0.34	5.67	17.09	**(99)**
Upper limits for natural baseline Concentrations in Taiwan	150.00	--	50.00	--	50.00	--	20.00	60.00	--	0.30	20.00	40.00	**(101)**
Average shale values (ASVs)	95.00	80,000.00	68.00	47,200.00	45.00	850.00	19.00	90.00	0.10	0.30	19.00	20.00	**(103)**

Supplementary Table 4: Compare the seasonal means of HM contents in the current study with different sediment quality guidelines (SQGs).

**Area & SQGs**	**Zn**	**Cr**	**Cu**	**Cd**	**Ni**	**Pb**
**Current Study**	**116.52**	**76.70**	**38.21**	**ND**	**24.44**	**ND**
Taiwan EPA lower limit	14	76	50	1	24	48
Taiwan EPA upper limit	384	233	157	3	80	161
CCME ISQG	123	37	36	1	----	35
CCME PEL	315	90	197	4	----	91
NOAA ERL	150	81	34	1	21	47
NOAA ERM	410	370	270	10	52	218
ANZECC & ARMCANZ low	200	80	95	2	21	50
ANZECC & ARMCANZ high	410	370	270	10	52	220
